# Tris(4-azidophenyl)methanol – a novel and multifunctional thiol protecting group†

**DOI:** 10.1039/d2ra05997e

**Published:** 2023-01-17

**Authors:** Xujun Qiu, Julian Brückel, Christoph Zippel, Martin Nieger, Frank Biedermann, Stefan Bräse

**Affiliations:** a Institute of Organic Chemistry (IOC), Karlsruhe Institute of Technology (KIT) Fritz-Haber-Weg 6 76131 Karlsruhe Germany braese@kit.edu (+49)-721-6084-2903; b Department of Chemistry, University of Helsinki P. O. Box 55 (A. I. Virtasen aukio 1) 00014 Finland; c Karlsruhe Institute of Technology (KIT), Institute of Nanotechnology (INT) Hermann-von-Helmholtz-Platz 1, 76344 Eggenstein-Leopoldshafen Germany; d Institute of Biological and Chemical Systems – Functional Molecular Systems (IBCS-FMS), Karlsruhe Institute of Technology (KIT) Hermann-von-Helmholtz-Platz 1, 76344 Eggenstein-Leopoldshafen Germany

## Abstract

The novel tris(4-azidophenyl)methanol, a multifunctionalisable aryl azide, is reported. The aryl azide can be used as a protecting group for thiols in peptoid synthesis and can be cleaved under mild reaction conditions *via* a Staudinger reduction. Moreover, the easily accessible aryl azide can be functionalised *via* copper-catalysed cycloaddition reactions, providing additional opportunities for materials chemistry applications.

## Abstract 2

The novel tris(4-azidophenyl)methanol, a multifunctionalizable aryl azide, is reported.

## Graphical abstract

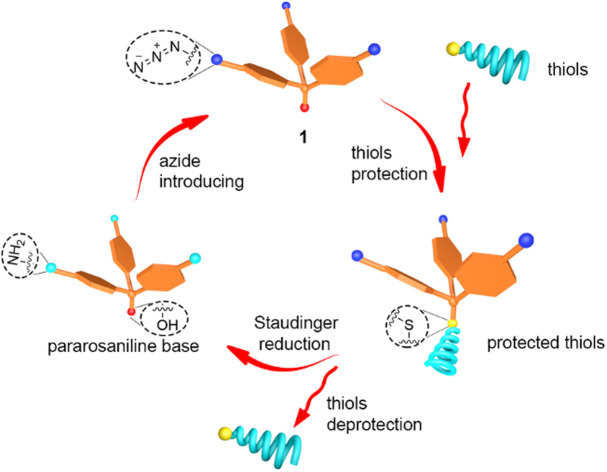

Organic azides are a class of nitrogen-rich compounds with extraordinarily versatile chemistry. The azido group readily undergoes functional group transformations such as 1,3-dipolar cycloaddition,^1–6^ Staudinger ligation,^7–12^ or C–H bond amination.^13–16^ The facile access to organic azides and the excellent yield and selectivity of their reactions has made them highly desirable for their potential applications in various fields, including organic and bioorganic chemistry,^17–21^ material science,^22–24^ and supramolecular chemistry.^25–27^

Aryl azides, in particular, have emerged as functional intermediates with diverse applications, among which the most well-known application is the copper(i)-catalysed azide–alkyne cycloaddition (CuAAC) click reaction.^28^ The copper(i) catalysed cycloaddition of aryl azides with alkynes allows the construction of triazole moieties, which were applied in constructing porous organic polymers^29–35^ and for the functionalisation of surfaces.^36–38^ Another imperative application of aryl azides is the Staudinger reaction, an integral approach to synthesising amides in the biosystem, such as peptide synthesis,^39^ bioactive molecular labelings,^40^ and cell engineering.^41^ The Staudinger reduction of aryl azides by applying phosphines is commonly used to prepare anilines, where the aryl azide serves as a stable precursor for amines.^42,43^

Inspired by the versatile azides, we report a multifunctional and spatially oriented aryl azide, which can be converted to triazoles *via* cycloaddition reaction and utilised as a protecting group for thiols in peptoid synthesis.

The novel aryl azide, tris(4-azidophenyl)methanol (1, Fig. 1), was synthesised according to a modified procedure by Kutonova *et al.*^44^ The arylamine was first diazotised with *tert*-butyl nitrite in the presence of *p*-toluenesulfonic acid, followed by the reaction with sodium azide in a one-pot procedure (Scheme 1).

**Fig. 1:**
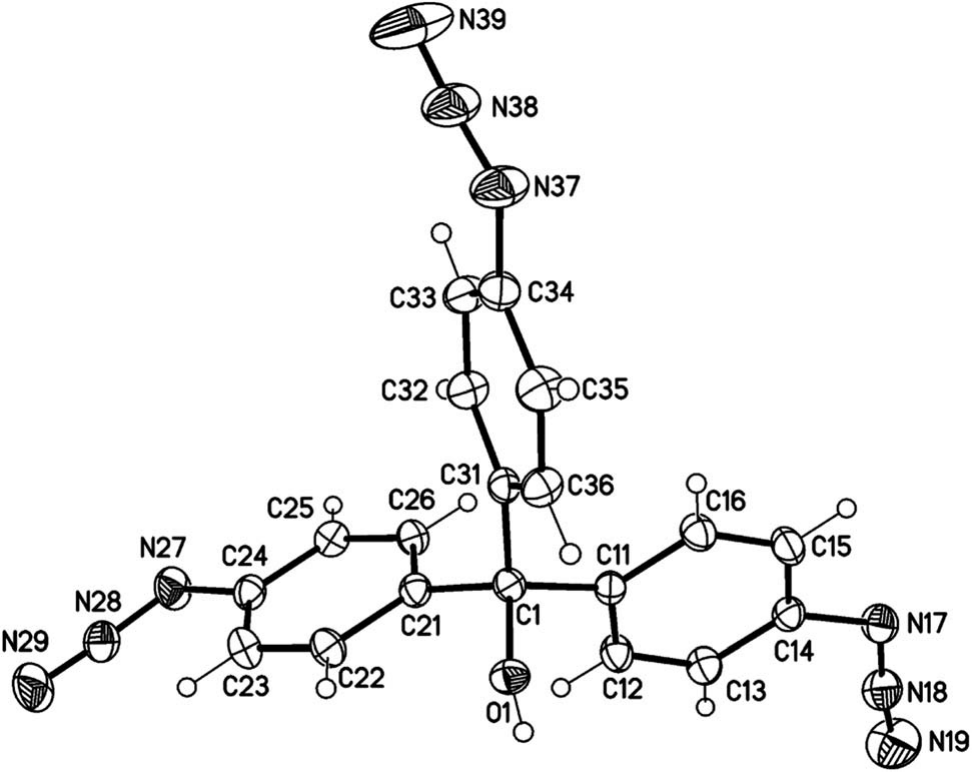
Molecular structure of aryl azide 1 (displacement parameters are drawn at a 30% probability level).

**Scheme 1:**
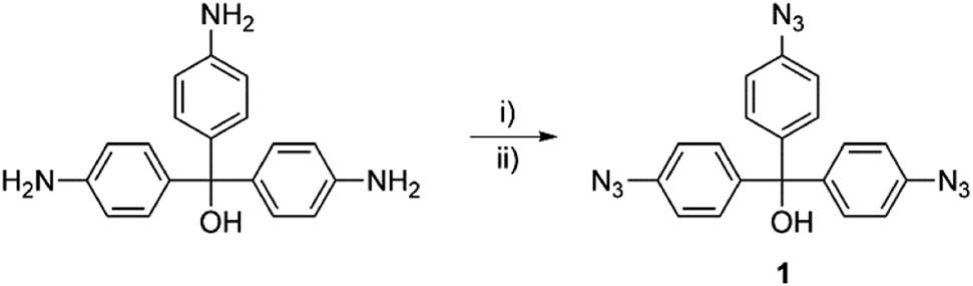
Syntheses of the multifunctional aryl azide 1. Reagents and conditions: (i) *p*-TsOH·H_2_O (4.50 equiv.), (CH_3_)_3_CONO (4.50 equiv.), CH_3_CN/H_2_O (2 : 1), r.t.; (ii) NaN_3_ (9.00 equiv.), H_2_O, r.t., (2 steps: 99%).

A set of cycloaddition reactions were performed utilising Cu(i) as catalyst to assess the potential functionalities of the synthesised aryl azide 1 (Scheme 2). The active hydroxyl group can be attached to other functional systems, such as the modification of the lipophilicity or the introduction of the other functional groups leading to a more sophisticated system.

**Scheme 2:**
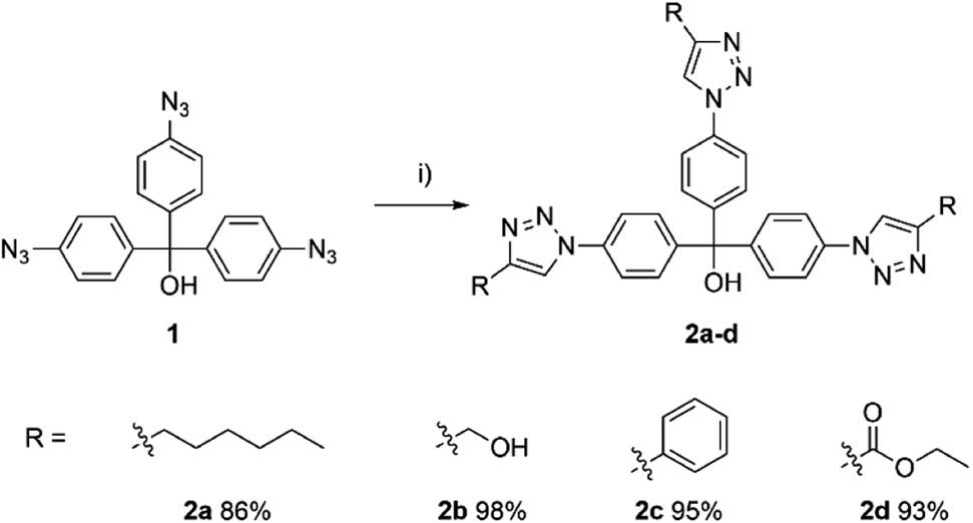
Aryl azide 1 based cycloaddition reaction. Reagents and conditions: (i) alkyne (3.00 equiv.), CuSO_4_·5H_2_O (0.30 equiv.), sodium ascorbate (0.90 equiv.), EtOH/H_2_O (1 : 1), 70 °C, 16 h.

Triphenylmethane and its derivatives, such as 4-methoxytrityl and 4-methyltrityl, were reported as excellent protecting groups for thiols.^45^ To evaluate the application of the newly discovered aryl azide 1, we explored a new strategy for protecting thiols with aryl azide 1, accomplishing deprotection under mild reaction conditions, in which the thioether bond was cleaved by a Staudinger reduction (Fig. 2).

**Fig. 2:**
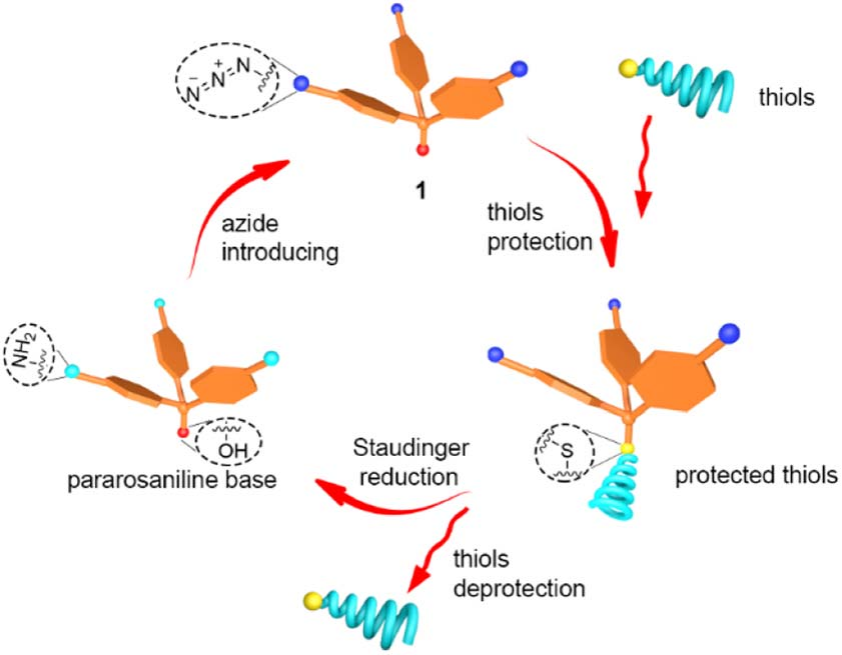
Schematic diagram of the thiols protecting and deprotecting process.

The studied thiols were first protected with aryl azide 1. The reaction was carried out either in TFA or in a TFA/CHCl_3_ mixture, which led to the desired products 3a–f in excellent yield (Scheme 3).

**Scheme 3:**
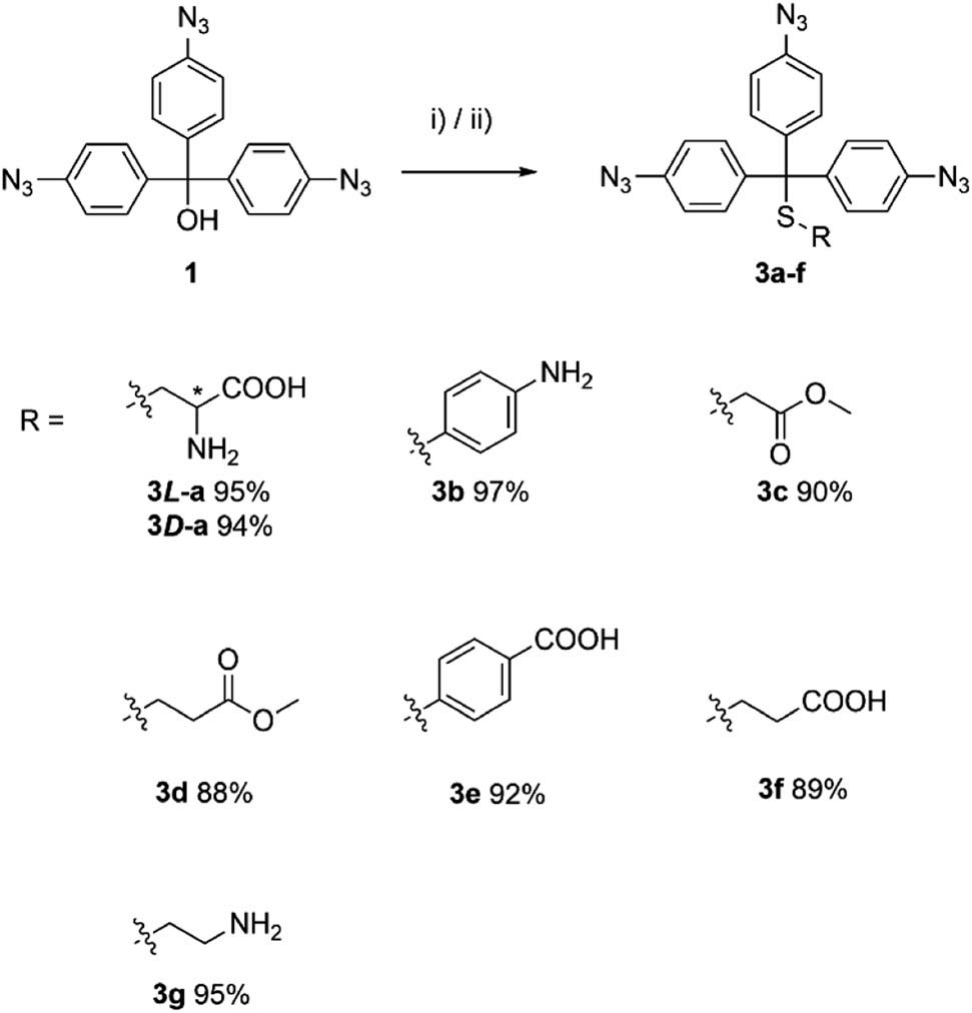
Syntheses of the aryl azide 1 protected thiols. Reagents and conditions: (i) thiol (1.00 equiv.), TFA, r.t., 2 h; or (ii) TFA/CHCl_3_, r.t., 2 h.

The deprotection was based on the Staudinger reduction, where the aryl azide-protected thiols were treated with trimethyl phosphine in THF/1 M HCl mixtures for 5 min at room temperature. The first step of the deprotection process involves the reduction of the azide groups to amines by reaction with trimethyl phosphine. The positive mesomeric effect (+M effect) renders the thioether bond labile, thus facilitating the cleavage of the thiols group under acidic conditions. This deprotection process based on aryl azide 1 is promising to protect thiols in solid-state synthesis.

We then utilised aryl azide 1 as protecting group for thiols in peptoid synthesis (see ESI† for details). As shown in Scheme 4, Fmoc-Phe-OH was first loaded to the polystyrene resin 5, then deprotected from the Fmoc group to obtain amine 6. After acetylation with 2-bromoacetic acid, the secondary amines 8a–e were formed by reacting primary amines with acetylated resin 7. By reacting 3f with 8a–e, a series of short peptoids 9a–e with aryl azide protected thiols were synthesised. Following the described procedure, the peptoid–peptide hybrids 14a–c were synthesized. In contrast to the peptoids 9a–e, the protected aminothiol was not incorporated at the end of the chain, but rather in the middle. After elongation, peptoids with a protected aminothiol as building block were obtained. In the case of 16f, the Fmoc-protecting group of the terminal amino acid was removed using common deprotection conditions, showing that the azide protecting group remains intact under the applied basic conditions.

**Scheme 4:**
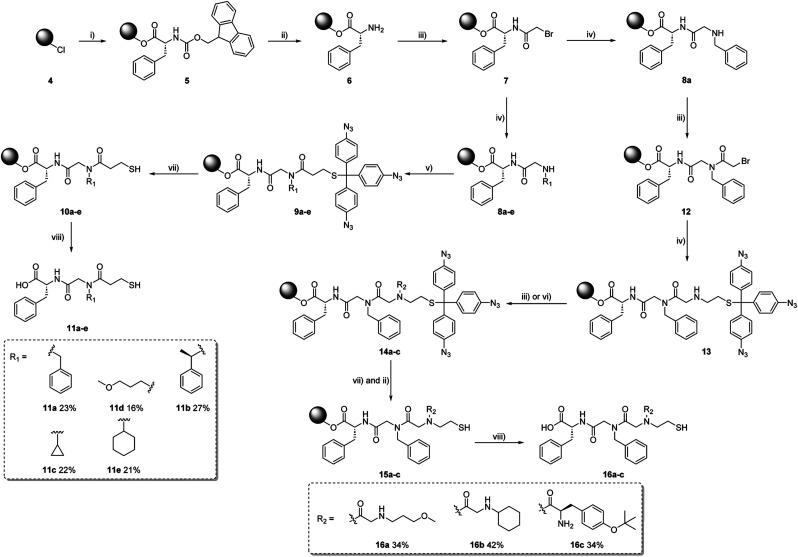
Peptoid synthesis. Reagents and conditions: (i) Fmoc-Phe-OH, DIPEA, NMP, r.t., 4 h; (ii) 20% piperidine in DMF, r.t., 3 × 5 min; (iii) bromoacetic acid, DIC, DMF, r.t., 0.5 h; (iv) primary amines, DMF, r.t., 1 h; (v) 3f, DIC, DMF, r.t., 4 h; (vi) amino acid, HOBt, DIC, DMF, r.t., 4 h; (vii) PMe_3_ in THF/1 M HCl, r.t., 5 min; (viii) 33% HFIP in DCM, r.t, 2 × 1 h.

The deprotection and cleavage process of the peptoids involves two orderly steps: the aryl azide protecting group was first deprotected using trimethyl phosphine in a THF/1 M HCl mixture within 5 min at room temperature to afford 10a–e and 15a–c. Afterward, the final peptoids (11a–e and 16a–c) were cleaved from the resin using a 33% solution of hexafluoro-2-propanol (HFIP) in dichloromethane, the successful synthesis was confirmed by LC-MS and ESI-MS.

In conclusion, a novel multifunctional aryl azide was efficiently synthesised in a one-pot reaction from a commercially available pararosaniline base. Thereby, a new strategy for protecting thiols in peptoid synthesis with the aryl azide 1 was established, for which deprotection can be accomplished by a Staudinger reduction under acid catalysis. Moreover, the spacious aryl azide can be functionalised by cycloaddition reaction, which provides additional options for use in materials chemistry.

## Conflicts of interest

No conflicts of interest to declare.

## Supplementary Material

RA-013-D2RA05997E-s001

RA-013-D2RA05997E-s002
