# Supplementary material for: Tris(4-azidophenyl)methanol – a novel and multifunctional thiol protecting group
Source: RSC Adv. 2023 Jan 17;13(4):2483–6. doi: 10.1039/d2ra05997e (PMC9844210; doi:10.1039/d2ra05997e)
Supplement: RA-013-D2RA05997E-s001 [file RA-013-D2RA05997E-s001.pdf]

Electronic Supplementary Material (ESI) for RSC Advances.
This journal is © The Royal Society of Chemistry 2022

Supporting Information

Tris(4-azidophenyl)methanol - a novel and multifunctional thiol protecting group

Xujun Qiu,[a] Julian Brückel,[a] Christoph Zippel,[a] Martin Nieger,[b] Frank Biedermann[c] and Stephan Bräse[a,d]*

[a] Institute of Organic Chemistry (IOC), Karlsruhe Institute of Technology (KIT), Fritz-Haber-Weg 6, 76131 Karlsruhe, Germany. Phone: (+49)-721-6084-2903; E-mail: braese@kit.edu.

[b] Department of Chemistry, University of Helsinki, P.O. Box 55 (A. I. Virtasen aukio 1), 00014 University of Helsinki, Finland.

[c] Karlsruhe Institute of Technology (KIT), Institute of Nanotechnology (INT), Hermann-von-Helmholtz-Platz 1, 76344 Eggenstein-Leopoldshafen, Germany.

[d] Institute of Biological and Chemical Systems – Functional Molecular Systems (IBCS-FMS), Karlsruhe Institute of Technology (KIT), Hermann-von-Helmholtz-Platz 1, 76344 Eggenstein-Leopoldshafen, Germany.

* Corresponding author

Table of Contents

1. **Additional Information** **1**

2. **General Remarks** **1**

3. **Synthetic Procedures** **4**

4. **Azide deprotection** **11**

5. **General procedures for peptoids synthesizing** **11**

6. **NMR Spectra** **12**

7. **X-Ray Diffractometry** **24**

8. **References** **25**

1. Additional Information

Safety precautions for working with azides compounds, please see [1, 2].

2. General Remarks

Materials and Methods

The starting materials, solvents, and reagents were purchased from ABCR, ACROS, ALFA AESAR, APOLLO SCIENTIFIC, CARBOLUTION, CHEMPUR, FLUKA, FLUOROCHEM, MERCK, RIEDEL-DE HAËN, SIGMA ALDRICH, STREM, TCI, or THERMO FISHER SCIENTIFIC and used without further purification unless stated otherwise.

Solvents of technical quality were purified by distillation or with the solvent purification system MB SPS5 (acetonitrile, dichloromethane, diethyl ether) from MBRAUN. Solvents of *p.a.* quality were purchased from ACROS, FISHER SCIENTIFIC, SIGMA ALDRICH, Roth, or RIEDEL-DE HAËN and were used without further purification.

For reactions, flat-bottom crimp neck vials from CHROMAGLOBE with an aluminum crimp cap were used.

Solvents were evaporated under reduced pressure at 45 °C using a rotary evaporator. For solvent mixtures, each solvent was measured volumetrically.

Flash column chromatography was performed using MERCK silica 60 (0.040 × 0.063 mm, 230–400 mesh ASTM) and quartz sand (glowed and purified with hydrochloric acid).

Reaction Monitoring

All reactions were monitored by thin-layer chromatography (TLC) using silica-coated aluminum plates (MERCK, silica 60, F254). UV active compounds were detected with a UV lamp at 254 nm and 366 nm excitation.

GC-MS (gas chromatography-mass spectrometry) measurements were performed on an AGILENT TECHNOLOGIES model 6890N (electron impact ionization), equipped with an AGILENT 19091S-433 column (5% phenyl methyl siloxane, 30 m, 0.25 µm) and a 5975B VL MSD detector with a turbopump. Helium was used as a carrier gas.

Nuclear Magnetic Resonance Spectroscopy (NMR)

NMR spectra were recorded on a BRUKER Avance 400 NMR instrument at 400 MHz for ^{1}H NMR, 101 MHz for ^{13}C NMR, or a BRUKER Avance 500 NMR instrument at 500 MHz for ^{1}H NMR, 126 MHz for ^{13}C NMR.

The NMR spectra were recorded at room temperature in deuterated solvents acquired from EURISOTOP, SIGMA ALDRICH, or DEUTERO. The chemical shift δ is displayed in parts per million [ppm] and the references used were the ^{1}H and ^{13}C peaks of the solvents themselves:

d_1-chloroform ($CDCl_3$): 7.26 ppm for ^{1}H and 77.16 ppm for ^{13}C

d_6-dimethyl sulfoxide (DMSO-d_6): 2.50 ppm for ^{1}H and 39.52 ppm for ^{13}C

For the characterization of centrosymmetric signals, the signal's median point was chosen for multiplets in the signal range. The following abbreviations were used to describe the proton splitting pattern: d = doublet, t = triplet, m = multiplet, dd = doublet of doublet, ddd = doublet of doublet of doublet, dddd = doublet of doublet of doublet of doublet, dt = doublet of triplet. Absolute values of the coupling constants "*J*" are given in Hertz [Hz] in absolute value and decreasing order. Signals of the ^{13}C spectrum were assigned by distortionless enhancement by polarization transfer (DEPT) spectra DEPT90 and DEPT135 or phase edited heteronuclear single quantum coherence (HSQC) and was specified in the following way: + = primary or tertiary carbon atoms (positive phase), – = secondary carbon atoms (negative phase), C_q = quaternary carbon atoms (no signal).

Infrared Spectroscopy (IR)

The infrared spectra were recorded with a BRUKER Alpha P instrument. All samples were measured by attenuated total reflection (ATR). The positions of the absorption bands are given in wavenumbers $\tilde{\nu}$ in cm^{-1} and were measured in the range from 3600 cm^{-1} to 500 cm^{-1}.

Characterization of the absorption bands was done in dependence on the absorption strength with the following abbreviations: vs (very strong, 0−9%), s (strong, 10−39%), m (medium, 40−69%), w (weak, 70−89%), vw (very weak, 90−100%).

Mass Spectrometry (MS)

APCI (atmospheric pressure chemical ionization) and ESI (electrospray ionization) experiments were recorded on a Q-Exactive (Orbitrap) mass spectrometer (THERMO FISHER SCIENTIFIC, San Jose, CA, USA) equipped with a HESI II probe to record high resolution. The tolerated error is ±5 ppm of the molecular mass. The spectra were interpreted by molecular peaks $[M]^+$, peaks of protonated molecules $[M+H]^{+,}$ and characteristic fragment peaks and indicated with their mass-to-charge ratio (*m/z*).

Liquid chromatography-mass spectrometry (LC-MS)

Liquid chromatography-mass spectrometry (LC−MS) was performed using a THERMOFISHER UltiMate 3000 system containing a degasser, pump, autosampler, column compartment, and diode array detector coupled with an ISQ™ EM Single Quadrupole Mass Spectrometer system. The flow rate was 0.45 mL/min on a stationary KINETEX XB-C18 column (2.1 mm × 100 mm, 2.6 µm particle size).

Crystallographic Information

The single-crystal X-ray diffraction study was carried out on a Bruker D8 Venture diffractometer with PhotonII CPAD detector at 298(2) K using Cu-Kα radiation (λ = 1.54178 Å) or Mo-Kα radiation (λ = 0.71073 Å). Dual space methods (SHELXT) [G. M. Sheldrick, Acta Crystallogr. 2015, A71, 3-8] were used for structure solution, and refinement was carried out using SHELXL-2014 (full-matrix least-squares on F2) [G. M. Sheldrick, Acta Crystallogr. 2015, C71, 3-8]. Hydrogen atoms were refined using a riding model. Semi-empirical absorption corrections were applied.

3. Synthetic Procedures

Tris(4-azidophenyl)methanol (1)

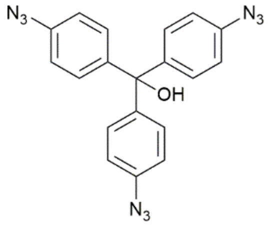

Tris(4-azidophenyl)methanol was synthesized according to the literature using a modified protocol[3]. p-Toluenesulfonic acid monohydrate (2.85 g, 15.0 mmol, 4.58 equiv.) was dissolved in acetonitrile/water (10/5.0 mL) at 21 °C. Once dissolved, tert-butyl nitrite (1.55 g, 15.0 mmol, 4.58 equiv.) was added, followed by pararosaniline base (1.00 g, 3.30 mmol, 1.00 equiv.) portion-wise. The reaction mixture was stirred for 3h at rt. After which, a solution of sodium azide (1.92 g, 29.5 mmol, 9.00 equiv.) in water (5.0 mL) was added dropwise. The resulting reaction mass was stirred for another 3 hours at 21 °C. After the complete conversion was achieved, the reaction mixture was extracted with ethyl acetate (3 × 20 ml). The combined organic layers were dried over sodium sulfate. After the removal of the solvent, the crude solid was purified by chromatography (EA/CH, 1:9), yielding tris(4-azidophenyl)methanol (1.24 g, 3.23 mmol, 99%) as brown-crystalline solid.

^{1}H NMR (500 MHz, Chloroform-*d*, ppm) δ = 7.25 – 7.21 (m, 6H, H_{Ar}), 7.00 – 6.95 (m, 6H, H_{Ar}), 2.71 (s, 1H, O*H*).

^{13}C NMR (126 MHz, Chloroform-*d*, ppm) δ = 143.3 (3C, C_{Ar}), 139.5 (3C, C_{Ar}), 129.4 (6C, C_{Ar}), 118.8 (6C, C_{Ar}), 81.2 (1C, *C*OH).

HRMS (ESI, $[M]^+$, $C_{19}H_{13}N_9O$) Calc. *m/z* = 383.1243, found *m/z* = 383.1232.

IR (ATR, ṽ) = 3491 (vs), 2117 (vs), 2108 (vs), 2096 (vs), 2078 (vs), 2029 (s), 1600 (vs), 1499 (vs), 1291 (vs), 1274 (vs), 1190 (s), 1184 (s), 1162 (s), 1121 (s), 1026 (s), 1011 (s), 835 (s), 806 (vs), 537 (s) cm^{-1}

Tris(4-(4-hexyl-1H-1,2,3-triazol-1-yl)phenyl)methanol (2a)

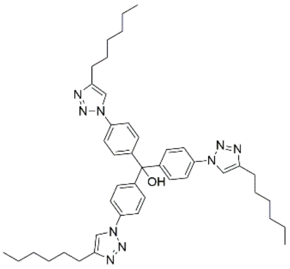

$CuSO_4 \cdot 5\,H_2O$ (9.74 mg, 0.04 mmol, 0.30 equiv.) and sodium ascorbate (23.2 mg, 0.12 mmol, 0.90 equiv.) were added to a solution of tris(4-azidophenyl)methanol (49.8 mg, 0.13 mmol, 1.00 equiv.) and 1-octin (43.0 mg, 0.39 mmol, 3.00 equiv.) in 1:1 ethanol/water (10 mL). The resulting yellowish cloudy suspension was stirred at 70 °C for 12 hours. Afterward, the reaction mixture was partitioned between water (10 mL)- ethyl acetate (20 mL).

The aqueous layer was extracted with additional ethyl acetate (2 x 10 mL). The combined organic layers were dried over sodium sulfate. After the removal of the solvent, the crude solid was purified by chromatography (DCM/MeOH, 20:1) resulting tris(4-(4-hexyl-1H-1,2,3-triazol-1-yl)phenyl)methanol (80.0 mg, 0.11 mmol, 86%) as light-yellow solid.

^{1}H NMR (500 MHz, Chloroform-*d*, ppm) δ = 7.71 (s, 3H, H_{Ar}), 7.69 (d, *J* = 8.5 Hz, 6H, H_{Ar}), 7.48 (d, *J* = 8.5 Hz, 6H, H_{Ar}), 3.93 (s, 1H, O*H*), 2.77 (t, *J* = 7.7 Hz, 6H, CH_2), 1.71 (p, *J* = 7.6 Hz, 6H, CH_2), 1.38 (t, *J* = 7.6 Hz, 6H, CH_2), 1.31 (h, *J* = 3.8 Hz, 12H, CH_2), 0.90 – 0.86 (m, 9H, CH_3)

^{13}C NMR (126 MHz, Chloroform-*d*, ppm) δ = 149.4 (3C, C_{Ar}), 146.3 (3C, C_{Ar}), 136.6 (3C, C_{Ar}), 129.2 (6C, C_{Ar}), 120.1 (6C, C_{Ar}), 118.7 (3C, C_{Ar}), 81.0 (1C, *C*OH), 31.6 (3C, CH_2), 29.3 (3C, CH_2), 28.9 (3C, CH_2), 25.6 (3C, CH_2), 22.6 (3C, CH_2), 14.1 (3C, CH_3).

HRMS (ESI, $[M+H]^+$, $C_{43}H_{56}N_9O$) Calc. *m/z* = 714.4530, found *m/z* = 714.4557.

IR (ATR, cm^{-1}) $\tilde{\nu}$ = 3139 (w), 3134 (w), 2953 (vs), 2925 (vs), 2855 (vs), 2360 (vs), 2343 (s), 2340 (s), 2337 (s), 2332 (s), 2331 (s), 2325 (m), 2323 (m), 2315 (w), 2313 (w), 1515 (vs), 1465 (m), 1456 (m), 1436 (m), 1409 (m), 1377 (w), 1363 (w), 1357 (w), 1354 (w), 1343 (m), 1334 (w), 1330 (w), 1321 (m), 1229 (s), 1220 (s), 1215 (m), 1206 (m), 1202 (w), 1198 (m), 1194 (m), 1179 (s), 1159 (m), 1121 (w), 1042 (vs), 988 (s), 831 (s), 798 (w), 668 (w), 593 (w), 559 (w), 556 (w), 554 (w), 552 (w), 549 (w), 547 (w), 545 (w), 404 (w), 401 (w) cm^{-1}.

(((hydroxymethanetriyl)tris(benzene-4,1-diyl))tris(1H-1,2,3-triazole-1,4-diyl))trimethanol (2b)

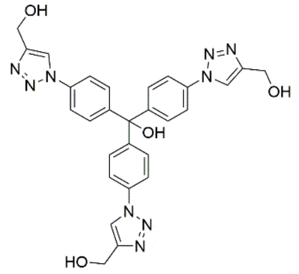

$CuSO_4 \cdot 5\ H_2O$ (9.74 mg, 0.04 mmol, 0.30 equiv.) and sodium ascorbate (23.2 mg, 0.12 mmol, 0.90 equiv.) were added to a solution of tris(4-azidophenyl)methanol (49.8 mg, 0.13 mmol, 1.00 equiv.) and 2-propyn-1-ol (21.9 mg, 0.39 mmol, 3.00 equiv.) in 1:1 ethanol/water (10 mL). The resulting yellowish cloudy suspension was stirred at 70 °C for 12 hours. After which, the reaction mixture was filtered, washed with water and ethanol resulting (((hydroxymethanetriyl)tris(benzene-4,1-diyl))tris(1H-1,2,3-triazole-1,4-diyl))trimethanol (70.0 mg, 0.13 mmol, 98%) as light-yellow solid.

^{1}H NMR (500 MHz, DMSO-d_6, ppm) δ = 8.67 (s, 3H, H_{Ar}), 7.90 (d, *J* = 8.3 Hz, 6H, H_{Ar}), 7.50 (d, *J* = 8.4 Hz, 6H, H_{Ar}), 6.99 (s, 1H, O*H*), 5.33 (d, *J* = 5.6 Hz, 3H, O*H*), 4.61 (d, *J* = 5.3 Hz, 6H, CH_2).

^{13}C NMR (126 MHz, DMSO-d_6, ppm) δ = 147.1 (3C, C_{Ar}), 135.6 (3C, C_{Ar}), 129.1 (6C, C_{Ar}), 121.0 (3C, C_{Ar}), 119.5 (6C, C_{Ar}), 79.8 (1C, *C*OH), 55.0 (3C, CH_2).

HRMS (ESI, $[M]^+$, $C_{28}H_{25}N_9O_4$) Calc. *m/z* = 551.2030, found *m/z* = 551.2802.

IR (ATR, cm^{-1}) $\tilde{\nu}$ = 3545 (br), 3391 (br), 3134 (vw), 2981 (s), 2359 (vs), 2342 (vs), 2340 (vs), 2336 (vs), 2332 (vs), 2331 (vs), 2325 (s), 2323 (s), 1514 (vs), 1482 (s), 1407 (s), 1228 (s), 1039 (s), 761 (s) cm^{-1}.

Tris(4-(4-phenyl-1H-1,2,3-triazol-1-yl)phenyl)methanol (2c)

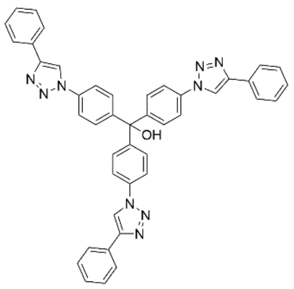

$CuSO_4 \cdot 5\ H_2O$ (9.74 mg, 0.04 mmol, 0.30 equiv.) and sodium ascorbate (23.2 mg, 0.12 mmol, 0.90 equiv.) were added to a solution of tris(4-azidophenyl)methanol (49.8 mg, 0.13 mmol, 1.00 equiv.) and ethynylbenzene (39.8 mg, 0.39 mmol, 3.00 equiv.) in 1:1 ethanol/water (10 mL). The resulting yellowish cloudy suspension was stirred at 70 °C for 12 hours. After which, the reaction mixture was filtered and washed with water and ethanol, yielding tris(4-(4-phenyl-1H-1,2,3-triazol-1-yl)phenyl)methanol (85.2 mg, 0.12 mmol, 95%) as light-yellow solid.

^1H NMR (500 MHz, DMSO-d_6, ppm) δ = 9.31 (s, 3H, H_{Ar}), 7.97 (t, *J* = 9.6 Hz, 12H, H_{Ar}), 7.58 (d, *J* = 7.1 Hz, 6H, H_{Ar}), 7.50 (t, *J* = 7.7 Hz, 6H, H_{Ar}), 7.39 (t, *J* = 7.4 Hz, 3H, H_{Ar}), 7.07 (s, 1H, O*H*).

^{13}C NMR (126 MHz, DMSO-d_6, ppm) δ = 147.3 (3C, C_{Ar}), 147.3 (3C, C_{Ar}), 135.5 (3C, C_{Ar}), 130.2 (3C, C_{Ar}), 129.2 (6C, C_{Ar}), 129.0 (6C, C_{Ar}), 128.3 (3C, C_{Ar}), 125.3 (6C, C_{Ar}), 119.6 (6C, C_{Ar}), 79.9 (1C, *C*OH).

HRMS (ESI, $[M+H]^+$, $C_{43}H_{32}N_9O$) Calc. *m/z* = 690.2652, found *m/z* = 690.2713.

IR (ATR, cm^{-1}) $\tilde{\nu}$ = 3292 (br), 3147 (vw), 2981 (s), 2361 (vs), 2359 (vs), 2343 (vs), 2340 (vs), 2337 (vs), 2332 (vs), 2331 (vs), 2323 (s), 1514 (vs), 1512 (vs), 1199 (s), 1060 (s), 1057 (s), 1051 (s), 1046 (vs), 1040 (vs), 1037 (vs) cm^{-1}.

Triethyl 1,1',1''-((hydroxymethanetriyl)tris(benzene-4,1-diyl))tris(1H-1,2,3-triazole-4-carboxylate) (2d)

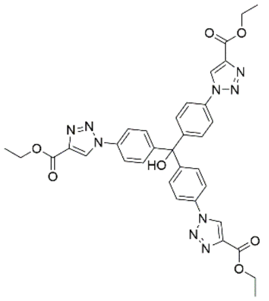

$CuSO_4 \cdot 5\ H_2O$ (9.74 mg, 0.04 mmol, 0.30 equiv.) and sodium ascorbate (23.2 mg, 0.12 mmol, 0.90 equiv.) were added to a solution of tris(4-azidophenyl)methanol (49.8 mg, 0.13 mmol, 1.00 equiv.) and ethyl propiolate (38.3 mg, 0.39 mmol, 3.00 equiv.) in 1:1 ethanol/water (10 mL). The resulting yellowish cloudy suspension was stirred at 70 °C for 12 hours. Afterward, the reaction mixture was partitioned between water (10 mL)- ethyl acetate (20 mL). The aqueous layer was extracted with additional ethyl acetate (2 x 10 mL). The combined organic layers were dried over sodium sulfate. After the removal of the solvent, the crude solid was purified by chromatography (DCM/MeOH, 20:1), yielding triethyl 1,1',1''-((hydroxymethanetriyl)tris(benzene-4,1-diyl))tris(1H-1,2,3-triazole-4-carboxylate) (82.0 mg, 0.12 mmol, 93%) as light-yellow solid.

^{1}H NMR (500 MHz, Chloroform-*d*, ppm) δ = 8.52 (s, 3H, H_{Ar}), 7.80 – 7.74 (m, 6H, H_{Ar}), 7.58 – 7.53 (m, 6H, H_{Ar}), 4.47 (q, *J* = 7.1 Hz, 6H, CH_2), 3.47 (s, 1H, O*H*), 1.44 (t, *J* = 7.1 Hz, 9H, CH_3).

^{13}C NMR (126 MHz, Chloroform-*d*, ppm) δ = 160.7 (3C, CO), 147.1 (3C, C_{Ar}), 141.2 (3C, C_{Ar}), 136.0 (3C, C_{Ar}), 129.5 (6C, C_{Ar}), 125.6 (3C, C_{Ar}), 120.9 (6C, C_{Ar}), 81.2 (1C, *C*OH), 61.8 (3C, CH_2), 14.5 (3C, CH_3).

HRMS (ESI, $[M+H]^+$, $C_{34}H_{32}N_9O_7$) Calc. *m/z* = 678.2346, found *m/z* = 678.2417.

IR (ATR, cm^{-1}) $\tilde{\nu}$ = 3452 (br), 3134 (m), 3076 (vw), 2981 (m), 2938 (w), 2916 (w), 2908 (w), 2906 (w), 2897 (w), 2361 (s), 2359 (s), 2343 (m), 2340 (m), 2337 (m), 2332 (m), 2331 (m), 2325 (m), 2323 (m), 2315 (w), 2313 (w), 2311 (w), 2309 (w), 1732 (vs), 1721 (vs), 1717 (vs), 1699 (s), 1695 (s), 1684 (m), 1675 (w), 1669 (w), 1557 (w), 1553 (w), 1549 (w), 1544 (w), 1541 (w), 1539 (w), 1533 (w), 1527 (w), 1515 (m), 1472 (w), 1464 (w), 1456 (w), 1445 (w), 1413 (w), 1394 (w), 1387 (w), 1370 (s), 1354 (m), 1343 (m), 1313 (w), 1298 (w), 1227 (vs), 1204 (s), 1200 (s), 1199 (s), 1194 (s), 1185 (s), 1174 (s), 1166 (s), 1146 (vs), 1096 (w), 1035 (vs), 1019 (s), 988 (w), 830 (m), 771 (m), 668 (w) cm^{-1}.

S-(tris(4-azidophenyl)methyl)-*L*/*D*-cysteine (3 *L*/*D*-a)

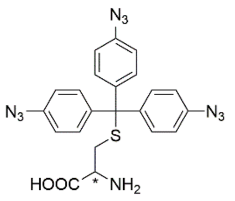

L/*D*-cysteine (96.9 mg, 0.80 mmol, 1.00 equiv.) was added to a solution of tris(4-azidophenyl)methanol (307 mg, 0.80 mmol, 1.00 equiv.) in trifluoroacetic acid (8.0 mL). The mixture was allowed to stir at room temperature for 2 hours. After the solvent removal, the residue was suspended in 10 ml diethyl ether, and naturized by sodium

acetate solution. The white precipitate was collected and washed with diethyl ether and cold water. After drying under the vacuo, S-(tris(4-azidophenyl)methyl)-*L*/*D*-cysteine (**3 *L*-a**: 370 mg, 0.76 mmol, 95%; **3 *D*-a**: 366 mg, 0.75 mmol, 94%) was obtained as a light pink powder.

For **3 *L*-a**:

^{1}H NMR (500 MHz, DMSO-d_6, ppm) δ = 7.40 – 7.31 (m, 6H, H_{Ar}), 7.18 – 7.06 (m, 6H, H_{Ar}), 2.98 (dd, *J* = 8.9, 4.1 Hz, 1H, C*H*), 2.50 – 2.44 (m, 1H, C*H*), 2.23 (dd, *J* = 11.7, 8.9 Hz, 1H, C*H*).

^{13}C NMR (126 MHz, DMSO-d_6, ppm) δ = 173.0 (1C, *C*O), 141.2 (3C, C_{Ar}), 137.8 (3C, C_{Ar}), 130.6 (6C, C_{Ar}), 118.8 (6C, C_{Ar}), 64.3 (1C, *C*S), 54.2 (1C, *C*N), 22.6 (1C, CH_2).

HRMS (ESI, $[M]^+$, $C_{22}H_{18}N_{10}O_2S$) Calc. *m/z* = 486.1335, found *m/z* = 486.2059.

IR (ATR, cm^{-1}) $\tilde{\nu}$ = 3033 (w), 3030 (w), 2989 (w), 2980 (w), 2971 (w), 2953 (w), 2948 (w), 2943 (w), 2939 (w), 2936 (w), 2931 (w), 2927 (w), 2925 (w), 2923 (w), 2920 (w), 2918 (w), 2361 (m), 2359 (m), 2343 (w), 2340 (w), 2337 (w), 2332 (w), 2331 (w), 2325 (w), 2323 (w), 2121 (vs), 2086 (vs), 2041 (w), 2036 (w), 1652 (w), 1647 (w), 1645 (w), 1637 (w), 1634 (w), 1627 (w), 1622 (w), 1619 (w), 1616 (w), 1600 (s), 1577 (m), 1575 (m), 1569 (m), 1557 (w), 1553 (w), 1550 (w), 1544 (w), 1538 (w), 1520 (w), 1500 (vs), 1464 (w), 1410 (w), 1404 (w), 1393 (w), 1389 (w), 1387 (w), 1281 (s), 1184 (w), 1120 (w), 827 (w), 797 (m) cm^{-1}.

For **3 *D*-a**:

^{1}H NMR (500 MHz, DMSO-d_6, ppm) δ = 7.34 – 7.31 (m, 6H, H_{Ar}), 7.10 – 7.08 (m, 6H, H_{Ar}), 2.98 (dd, *J* = 9.0, 4.4 Hz, 1H, C*H*), 2.55 – 2.50 (m, 1H, C*H*), 2.30 (dd, *J* = 12.1, 8.9 Hz, 1H, C*H*).

^{13}C NMR (126 MHz, DMSO-d_6, ppm) δ = 141.0 (3C, C_{Ar}), 137.9 (3C, C_{Ar}), 130.6 (6C, C_{Ar}), 118.9 (6C, C_{Ar}), 64.5 (1C, *C*S), 53.8 (1C, *C*N), 21.5 (1C, CH_2).

HRMS (ESI, $[M]^+$, $C_{22}H_{18}N_{10}O_2S$) Calc. *m/z* = 486.1335, found *m/z* = 486.2059.

IR (ATR, cm^{-1}) $\tilde{\nu}$ = 3032 (w), 2981 (m), 2971 (w), 2959 (w), 2953 (w), 2947 (w), 2945 (w), 2942 (w), 2930 (w), 2922 (w), 2918 (w), 2361 (m), 2359 (m), 2342 (w), 2340 (w), 2337 (w), 2332 (w), 2331 (w), 2119 (vs), 2084 (vs), 2037 (w), 1651 (w), 1634 (m), 1626 (m), 1623 (m), 1618 (m), 1616 (m), 1600 (s), 1575 (s), 1559 (m), 1554 (m), 1545 (w), 1541 (w), 1538 (w), 1533 (w), 1528 (w), 1525 (w), 1520 (w), 1499 (vs), 1435 (w), 1409 (m), 1404 (m), 1393 (m), 1389 (m), 1387 (m), 1279 (vs), 1185 (m), 1130 (m), 1123 (m), 827 (w), 796 (m) cm^{-1}.

4-((Tris(4-azidophenyl)methyl)thio)aniline (3b)

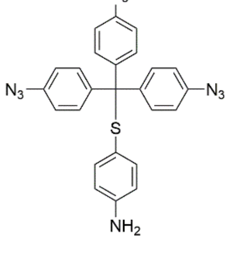

4-Aminothiophenol (100 mg, 0.80 mmol, 1.00 equiv.) was added to a solution of tris(4-azidophenyl)methanol (307 mg, 0.80 mmol, 1.00 equiv.) and trifluoroacetic acid (2.0 mL) in chloroform (10 mL). The mixture was allowed to stir at room temperature for 2 hours. After the solvent removal, the residue was suspended in 10 ml ethyl acetate, and naturized by sodium acetate solution. The aqueous layer was extracted with additional ethyl acetate (2 x 10 mL). The combined organic layers were dried over sodium sulfate. After the removal of the solvent, the crude solid was purified by chromatography (EtOAc/CH, 3:7), yielding 4-((tris(4-azidophenyl)methyl)thio)aniline (381 mg, 0.78 mmol, 97%) as gray solid.

^{1}H NMR (500 MHz, Chloroform-*d*, ppm) δ = 7.32 – 7.28 (m, 6H, H_{Ar}), 6.91 – 6.87 (m, 6H, H_{Ar}), 6.74 (d, *J* = 8.5 Hz, 2H, H_{Ar}), 6.39 – 6.35 (m, 2H, H_{Ar}), 3.73 (s, 2H, NH_2).

^{13}C NMR (126 MHz, Chloroform-*d*, ppm) δ = 147.7 (1C, C_{Ar}), 141.4 (2C, C_{Ar}), 138.6 (2C, C_{Ar}), 138.4 (3C, C_{Ar}), 131.4 (6C, C_{Ar}), 120.2 (1C, C_{Ar}), 118.4 (6C, C_{Ar}), 114.9 (3C, C_{Ar}), 69.3 (1C, *C*S).

HRMS (ESI, [M+H]$^+$, $C_{25}H_{19}N_{10}S$) Calc. *m/z* = 491.1437, found *m/z* = 491.1507.

IR (ATR, cm^{-1}) $\tilde{\nu}$ = 3473 (w), 3375 (m), 3235 (w), 3205 (w), 3028 (w), 2981 (m), 2361 (s), 2359 (s), 2343 (s), 2340 (s), 2337 (m), 2332 (m), 2331 (m), 2325 (m), 2323 (m), 2115 (vs), 2113 (vs), 2099 (vs), 2080 (vs), 2079 (vs), 2027 (m), 2022 (m), 2019 (m), 2015 (m), 2011 (m), 2007 (m), 1617 (m), 1594 (s), 1575 (m), 1491 (vs), 1276 (vs), 1185 (m), 1176 (m), 820 (w) cm^{-1}.

Methyl 2-((tris(4-azidophenyl)methyl)thio)acetate (3c)

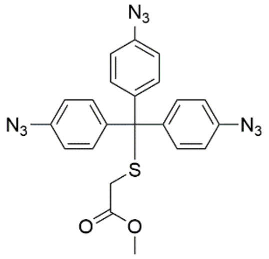

Methyl thioglycolate (84.9 mg, 0.80 mmol, 1.00 equiv.) was added to a solution of tris(4-azidophenyl)methanol (307 mg, 0.80 mmol, 1.00 equiv.) and trifluoroacetic acid (2.0 mL) in chloroform (10 mL). The mixture was allowed to stir at room temperature for 2 hours. After the solvent removal, the residue was suspended in 10 ml ethyl acetate, and naturized by sodium acetate solution. The aqueous layer was extracted with additional ethyl acetate (2 x 10 mL). The combined organic layers were dried over sodium sulfate. After the removal of the solvent, the crude solid was purified by chromatography (EtOAc/CH, 2:8), yielding methyl 2-((tris(4-azidophenyl)methyl)thio)acetate (339 mg, 0.72 mmol, 90%) as a colorless oil.

^{1}H NMR (400 MHz, Chloroform-*d*, ppm) δ = 7.40 – 7.33 (m, 6H, H_{Ar}), 7.00 – 6.93 (m, 6H, H_{Ar}), 3.60 (s, 3H, CH_3), 2.96 (s, 2H, CH_2).

^{13}C NMR (101 MHz, Chloroform-*d*, ppm) δ = 169.8 (1C, *C*O), 140.5 (3C, C_{Ar}), 139.2 (3C, C_{Ar}), 130.9 (6C, C_{Ar}), 119.0 (6C, C_{Ar}), 65.9 (1C, *C*S), 52.6 (1C, CH_3), 34.6 (1C, CH_2).

HRMS (ESI, $[M+H]^+$, $C_{22}H_{18}N_9O_2S$) Calc. *m/z* = 472.1226, found *m/z* = 472.2047.

IR (ATR, cm^{-1}) $\tilde{\nu}$ = 3480 (w), 3384 (w), 3237 (w), 3211 (w), 3059 (vw), 3030 (w), 2992 (vw), 2980 (w), 2950 (m), 2361 (s), 2359 (s), 2342 (s), 2340 (s), 2337 (s), 2331 (s), 2116 (vs), 2115 (vs), 2102 (vs), 2081 (vs), 2035 (s), 2031 (s), 2027 (s), 2023 (s), 2019 (s), 2015 (s), 2012 (s), 2008 (s), 2003 (s), 2000 (s), 1996 (s), 1992 (m), 1988 (m), 1736 (vs), 1600 (vs), 1575 (s), 1498 (vs), 1495 (vs), 1435 (s), 1275 (vs), 1233 (s), 1225 (s), 1222 (m), 1218 (m), 1214 (m), 1210 (m), 1206 (s), 1202 (s), 1186 (vs), 1165 (s), 1162 (s), 1153 (s), 1150 (s), 1146 (s), 1142 (s), 1138 (s), 1130 (vs), 1128 (vs), 1123 (vs), 1013 (s), 825 (s), 822 (s), 819 (s), 815 (s), 810 (s), 796 (vs), 549 (s), 541 (s), 537 (s), 533 (s), 530 (s), 518 (s), 411 (vs), 404 (vs), 400 (vs) cm^{-1}.

Methyl 3-((tris(4-azidophenyl)methyl)thio)propanoate (3d)

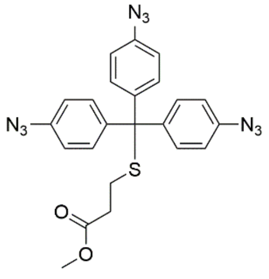

Methyl 3-mercaptopropionate (96.1 mg, 0.80 mmol, 1.00 equiv.) was added to a solution of tris(4-azidophenyl)methanol (307 mg, 0.80 mmol, 1.00 equiv.) and trifluoroacetic acid (2.0 mL) in chloroform (10 mL). The mixture was allowed to stir at room temperature for 2 hours. After the solvent removal, the residue was suspended in 10 ml ethyl acetate and naturized by sodium acetate solution. The aqueous layer was extracted with additional ethyl acetate (2 x 10 mL). The combined organic layers were dried over sodium sulfate. After the removal of the solvent, the crude solid was purified by chromatography (EtOAc/CH, 2:8), yielding methyl 3-((tris(4-azidophenyl)methyl)thio)propanoate (342 mg, 0.70 mmol, 88%) as a light-yellow solid.

^{1}H NMR (400 MHz, Chloroform-*d*, ppm) δ = 7.40 – 7.32 (m, 6H, H_{Ar}), 7.00 – 6.92 (m, 6H, H_{Ar}), 3.66 (s, 3H, CH_3), 2.46 – 2.38 (m, 2H, CH_2), 2.36 – 2.27 (m, 2H, CH_2).

^{13}C NMR (101 MHz, Chloroform-*d*, ppm) δ = 172.2 (1C, *C*O), 141.1 (3C, C_{Ar}), 138.9 (3C, C_{Ar}), 130.9 (6C, C_{Ar}), 118.9 (6C, C_{Ar}), 65.5 (1C, *C*S), 52.0 (1C, CH_3), 33.2 (1C, CH_2), 27.1 (1C, CH_2).

HRMS (ESI, $[M-H]^+$, $C_{23}H_{18}N_9O_2S$) Calc. *m/z* = 484.1382, found *m/z* = 484.1297.

IR (ATR, cm^{-1}) $\tilde{\nu}$ = 2287 (s), 2280 (s), 2276 (s), 2272 (s), 2234 (s), 2228 (s), 2225 (vs), 2219 (vs), 2213 (vs), 2209 (vs), 2204 (vs), 2198 (vs), 2194 (vs), 2188 (vs), 2183 (vs), 2178 (vs), 2173 (vs), 2167 (s), 2157 (s), 1657 (s), 1654 (s), 1649 (s), 1642 (s), 1638 (s), 1632 (s), 1546 (s), 1543 (s), 1540 (s), 1535 (s), 1528 (s), 1526 (s), 1523 (s), 1518 (s), 1379 (s), 1375 (s), 1332 (s), 1328 (s), 1026 (s), 994 (s), 967 (s), 962 (s), 958 (s), 931 (s), 916 (s), 905 (s), 883 (s), 879 (vs), 873 (vs), 869 (vs), 864 (vs), 858 (vs), 854 (vs), 725 (s), 708 (s), 703 (s), 699 (s), 683 (s), 678 (s), 652 (s), 647 (s), 642 (s), 638 (s), 633 (s), 627 (s), 623 (vs), 618 (vs), 613 (vs), 608 (vs), 603 (vs), 598 (vs), 593 (vs), 586 (vs), 581 (vs), 576 (vs), 571 (vs), 566 (vs), 562 (vs), 557 (vs), 510 (s), 505 (vs), 500 (s), 495 (s), 489 (s), 484 (vs), 479

(vs), 473 (vs), 468 (vs), 463 (vs), 458 (vs), 453 (vs), 448 (s), 445 (s), 440 (vs), 435 (vs), 430 (vs), 425 (vs), 420 (vs), 415 (vs), 409 (vs), 404 (vs) cm^{-1}.

4-((Tris(4-azidophenyl)methyl)thio)benzoic acid (3e)

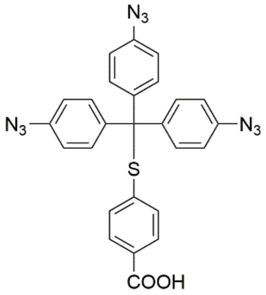

4-Mercaptobenzoic acid (123 mg, 0.80 mmol, 1.00 equiv.) was added to a solution of tris(4-azidophenyl)methanol (307 mg, 0.80 mmol, 1.00 equiv.) in trifluoroacetic acid (8.0 mL). The mixture was allowed to stir at room temperature for 2 hours. After the solvent removal, the residue was suspended in 10 ml ethyl acetate and naturized by sodium acetate solution. The aqueous layer was extracted with additional ethyl acetate (2 x 10 mL). The combined organic layers were dried over sodium sulfate. After the removal of the solvent, the crude solid was purified by chromatography (MeOH/DCM, 1:9), yielding 4-((tris(4-azidophenyl)methyl)thio)benzoic acid (382 mg, 0.74 mmol, 92%) as gray solid.

^{1}H NMR (500 MHz, DMSO-d_6, ppm) δ = 13.01 (s, 1H, COO*H*), 7.64 – 7.59 (m, 2H, H_{Ar}), 7.34 – 7.28 (m, 6H, H_{Ar}), 7.10 – 7.05 (m, 6H, H_{Ar}), 7.03 – 6.98 (m, 2H, H_{Ar}).

^{13}C NMR (126 MHz, DMSO-d_6, ppm) δ = 166.8 (1C, *C*O), 140.0 (3C, C_{Ar}), 139.7 (1C, C_{Ar}),138.4 (3C, C_{Ar}), 131.6 (2C, C_{Ar}), 130.9 (6C, C_{Ar}), 129.5 (1C, C_{Ar}),129.2 (2C, C_{Ar}),118.9 (6C, C_{Ar}), 69.2 (1C, *C*S).

HRMS (ESI, $[M]^+$, $C_{26}H_{17}N_9O_2S$) Calc. m/z = 519.1226, found m/z = 519.1384.

IR (ATR, cm^{-1}) $\tilde{\nu}$ = 3409 (vw), 3243 (w), 3213 (w), 3087 (vw), 3064 (vw), 3024 (vw), 2980 (m), 2926 (vw), 2887 (vw), 2850 (vw), 2662 (w), 2546 (w), 2416 (w), 2361 (s), 2359 (s), 2343 (m), 2340 (s), 2337 (m), 2332 (m), 2331 (m), 2323 (m), 2121 (vs), 2119 (vs), 2117 (vs), 2105 (vs), 2103 (vs), 2084 (vs), 2024 (m), 2020 (m), 2016 (m), 2014 (m), 2010 (m), 2008 (m), 2004 (m), 1684 (vs), 1594 (s), 1575 (s), 1500 (vs), 1498 (vs), 1495 (vs), 1418 (s), 1413 (s), 1293 (vs), 1281 (vs), 1275 (vs), 1245 (s), 1241 (s), 1216 (m), 1212 (m), 1186 (s), 1130 (s), 1014 (m), 805 (m), 789 (s), 765 (m) cm^{-1}.

3-((Tris(4-azidophenyl)methyl)thio)propanoic acid (3f)

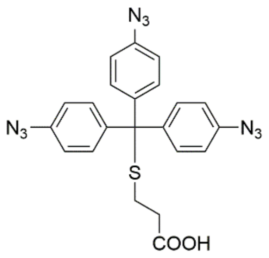

3-Mercaptopropionic acid (84.9 mg, 0.80 mmol, 1.00 equiv.) was added to a solution of tris(4-azidophenyl)methanol (307 mg, 0.80 mmol, 1.00 equiv.) in trifluoroacetic acid (8.0 mL). The mixture was allowed to stir at room temperature for 2 hours. After the solvent removal, the residue was suspended in 10 ml ethyl acetate and naturized by sodium acetate solution. The aqueous layer was extracted with additional ethyl acetate (2 x 10 mL). The combined organic layers were dried over sodium sulfate. After the removal of the solvent, the crude solid was purified by chromatography (MeOH/DCM, 1:9), yielding 3-((tris(4-azidophenyl)methyl)thio)propanoic acid (336 mg, 0.71 mmol, 89%) as gray solid.

^{1}H NMR (500 MHz, Chloroform-*d*, ppm) δ = 7.20 (d, *J* = 8.3 Hz, 6H, H_{Ar}), 6.76 (d, *J* = 8.2 Hz, 6H, H_{Ar}), 2.15 (d, *J* = 7.1 Hz, 2H, CH_2), 1.99 (d, *J* = 7.6 Hz, 2H, CH_2).

^{13}C NMR (126 MHz, Chloroform-*d*, ppm) δ = 180.0 (1C, *C*O), 141.2 (3C, C_{Ar}), 138.8 (3C, C_{Ar}), 130.8 (6C, C_{Ar}), 118.8 (6C, C_{Ar}), 65.4 (1C, *C*S), 36.5 (1C, *C*H_2), 28.6 (1C, *C*H_2).

HRMS (ESI, $[M]^+$, $C_{22}H_{17}N_9O_2S$) Calc. *m/z* = 471.1226, found *m/z* = 471.1963.

IR (ATR, cm^{-1}) $\tilde{\nu}$ = 3393 (br), 3241 (w), 3205 (vw), 3114 (vw), 3087 (vw), 3062 (vw), 3030 (w), 2981 (m), 2971 (m), 2931 (w), 2929 (w), 2359 (m), 2343 (m), 2340 (m), 2337 (m), 2332 (w), 2331 (w), 2117 (vs), 2102 (vs), 2082 (vs), 2027 (m), 1599 (s), 1574 (vs), 1572 (vs), 1498 (vs), 1398 (s), 1276 (vs), 1185 (s), 1130 (s), 1124 (s), 1014 (w), 826 (m), 796 (s), 537 (m), 533 (m) cm^{-1}.

3-((Tris(4-azidophenyl)methyl)thio)propanoic acid (3f)

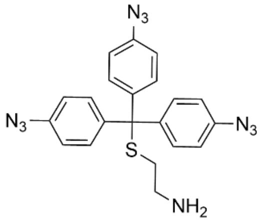

2-Aminoethanethiol hydrochloride (90.9 mg, 0.80 mmol, 1.00 equiv.) was added to a solution of tris(4-azidophenyl)methanol (307 mg, 0.80 mmol, 1.00 equiv.) and trifluoroacetic acid (8.0 mL). The mixture was allowed to stir at room temperature for 2 hours. After the solvent removal, the residue was suspended in 10 ml ethyl acetate, and naturized by sodium acetate solution. The aqueous layer was extracted with additional ethyl acetate (2 x 10 mL). The combined organic layers were dried over sodium sulfate. After the removal of the solvent, the crude solid was purified by chromatography (MeOH / DCM, 2:8) to obtain methyl 2-((tris(4-azidophenyl)methyl)thio)ethan-1-amine (330 mg, 0.75 mmol, 93%) was obtained as brown solid.

^{1}H NMR (400 MHz, Chloroform-*d*) δ = 7.40–7.29 (m, 6H, *H*Ar), 7.01–6.90 (m, 6H, *H*Ar), 2.52 (d, *J* = 6.0 Hz, 2H, CH_2), 2.47 (d, *J* = 6.2 Hz, 2H, CH_2), 2.34 (s, 2H, NH_2).

^{13}C NMR (126 MHz, Chloroform-*d*, ppm) δ = 140.3 (3C, C_{Ar}), 139.4 (3C, C_{Ar}), 130.7 (6C, C_{Ar}), 119.1 (6C, C_{Ar}), 66.2 (1C, *C*S), 38.6 (1C, CH_2), 29.1 (1C, CH_2).

HRESI-MS (m/z): 340.1303 [M-H_4N_7]$^+$, 314.1398 [M-$C_3H_6N_7$]$^+$. Due to the thermal instability of the compound, only fragments could be assigned.

IR (ATR, ṽ) = 2922 (s), 2920 (s), 2918 (s), 2916 (s), 2120 (vs), 2118 (vs), 2117 (vs), 2115 (vs), 2111 (vs), 2105 (vs), 2102 (vs), 2101 (vs), 2100 (vs), 2098 (vs), 2085 (vs), 2083 (vs), 2081 (vs), 2056 (s), 1686 (s), 1676 (s), 1674 (s), 1672 (s), 1671 (s), 1668 (s), 1662 (s), 1600 (s), 1500 (vs), 1498 (vs), 1495 (vs), 1278 (vs), 1200 (s), 1197 (s), 1184 (s), 1180 (s), 1129 (s), 795 (s) cm^{-1}.

4. Azide deprotection

Tris(4-azidophenyl)methanol protected compounds **3a-3g** (0.08 mmol) were added to a solution of 1 M Me_3P in THF: 1M HCl (9:1, 0.5 ml). The reaction mixture was stirred at room temperature for 5 minutes. The quantitative conversion of the deprotected compound was monitored by LC-MS

5. Synthesize of peptoids

For the synthesis of the peptide-peptoids hybrids the following general procedures (**GP**) were used.

Loading the resin with an amino acid (**GP 1**)

In a fritted syringe, the 2-chlorotrityl chloride modified polystyrene resin (125 mg, 200 µmol, 1.00 equiv.) was swollen in 2.0 mL dichloromethane by shaking at 21°C for at least 30 min. The solvent was removed. The Fmoc-protected amino acid Fmoc-Phe-OH (310 mg, 800 µmol, 4.00 equiv.) and diisopropylethylamine (DIPEA, 139 µL, 103 mg, 800 µmol, 4.00 equiv.) were dissolved in 2.0 mL *N*-methyl-2-pyrrolidone (NMP) and added to the resin. After shaking for at least 4 h, the solvent was removed and the resin was washed with 2.0 mL each of DMF, methanol, and dichloromethane.

Deprotection of Fmoc-protecting group (**GP 2**)

Deprotection of the Fmoc-protecting group was carried out using a solution of 20% piperidine in DMF 3.0 mL were added to the resin and let shaken for at least 5 min. The deprotection cocktail was removed and the procedure was repeated twice. The resin was washed with 2.0 mL each of DMF, methanol and dichloromethane.

Acetylation of the loaded resin (**GP 3**)

Acetylation of the resin was performed using diisopropylcarbodiimide (DIC, 250 µL, 1600 µmol, 8.00 equiv.) dissolved in a 1 M solution of bromoacetic acid (222 mg, 1600 µmol, 8.00 equiv.) in DMF. The mixture was

immediately added to the resin and let shaken for 30 min. The solvent was removed, and the resin was washed with 2.0 mL each of DMF, methanol and dichloromethane.

Substitution with an amine (**GP 4**)

The desired amine (1600 µmol, 8.00 equiv.) was dissolved in 2.0 mL DMF, added to the resin, and let shaken for at least 1 h. The solvent was removed, and the resin was washed with 2.0 mL each of DMF, methanol, and dichloromethane.

Coupling of an amino acid (**GP 5**)

The desired amino acid (800 µmol, 4.00 equiv.), 1-hydroxybenzotriazole (108 mg, 800 µmol, 4.00 equiv.) and diisopropylcarbodiimide (DIC, 125 µL, 800 µmol, 4.00 equiv.) were dissolved in 2.00 mL of DMF. The mixture was added to the resin and was let shaken for at least 4 h. The solvent was removed, and the resin was washed with each of DMF, methanol and dichloromethane.

Synthesis of peptoids (**11a-e**)

The first amino acid was attached to the resin according to **GP 1**. Deprotection of the Fmoc-protecting group was carried out following **GP 2**. Acetylation of the unprotected amino acid was performed according to **GP 3**. The subsequent substation with the desired amine was performed following **GP 4**. The azide-protected carboxylic acid **3f** (337mg, 800 µmol, 4.00 equiv.) and diisopropylcarbodiimide (DIC, 125 µL, 800 µmol, 4.00 equiv.) were dissolved in 2.00 mL DMF, added to the resin, and let shaken for at least 4 h. The solvent was removed and the last washing step after substitution was followed by washing with dichloromethane.

Synthesis of peptoids (**16a-b**)

The first amino acid was attached to the resin according to **GP 1**. Deprotection of the Fmoc-protecting group was carried out following **GP 2**. Acetylation of the unprotected amino acid was performed according to **GP 3**. The subsequent substation with the desired amine was performed following **GP 4**. Acetylation and substitution according to **GP 3** and **GP 4** was repeated twice until the desired tetramer was synthesized. The solvent was removed, and the resin was washed with each of DMF, methanol and dichloromethane.

Synthesis of peptoid (**16c**)

The first amino acid was attached to the resin according to **GP 1**. Deprotection of the Fmoc-protecting group was carried out following **GP 2**. Acetylation of the unprotected amino acid was performed according to **GP 3**. The subsequent substation with the desired amine was performed following **GP 4**. Acetylation and substitution according to **GP 3** and **GP 4** was repeated. Coupling of the amino acid was performed using **GP 5**. The deprotection of the Fmoc-protecting group was carried out according to **GP 2**. The solvent was removed, and the resin was washed with each of DMF, methanol and dichloromethane.

Peptoids deprotection

The loaded resin was incubated with a solution of 1 M Me_3P in THF: 1 M HCl (9:1, 2.0 ml). The reaction mixture was shanked carefully at room temperature for 5 minutes, then washed with DMF, methanol, and dichloromethane.

The loaded resin was washed three times with 2.00 mL of dichloromethane and then incubated twice with 2.5 mL of a 33% solution of hexafluoro-2-propanol (HFIP) in dichloromethane for 1 h each time. The resin was washed five times with 2.0 mL dichloromethane, and the solutions were combined. After evaporating the solvent under an air stream, the residue was dissolved in 5.0 mL acetonitrile/water (4:1) and lyophilized.

6. NMR Spectra

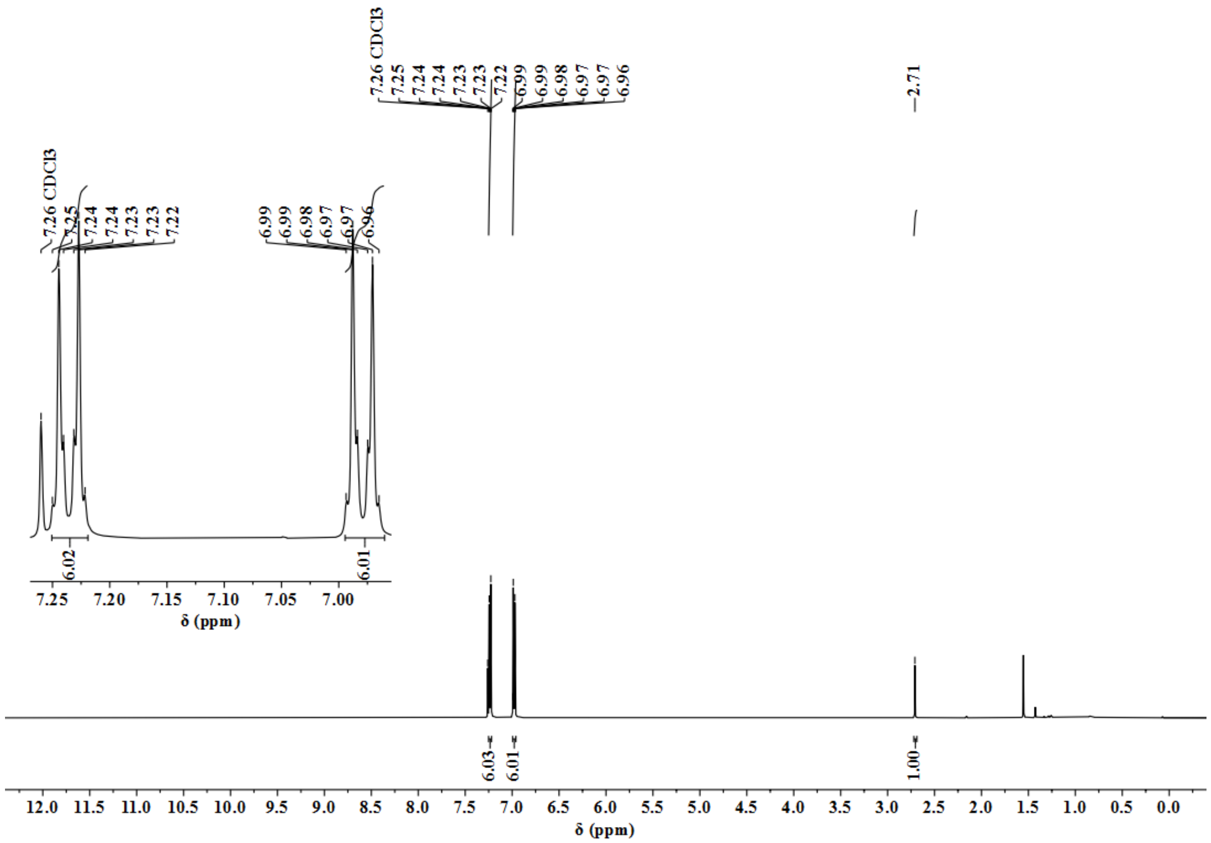

Figure S1. ^{1}H NMR –Tris(4-azidophenyl)methanol (**1**), 500 MHz, $CDCl_3$.

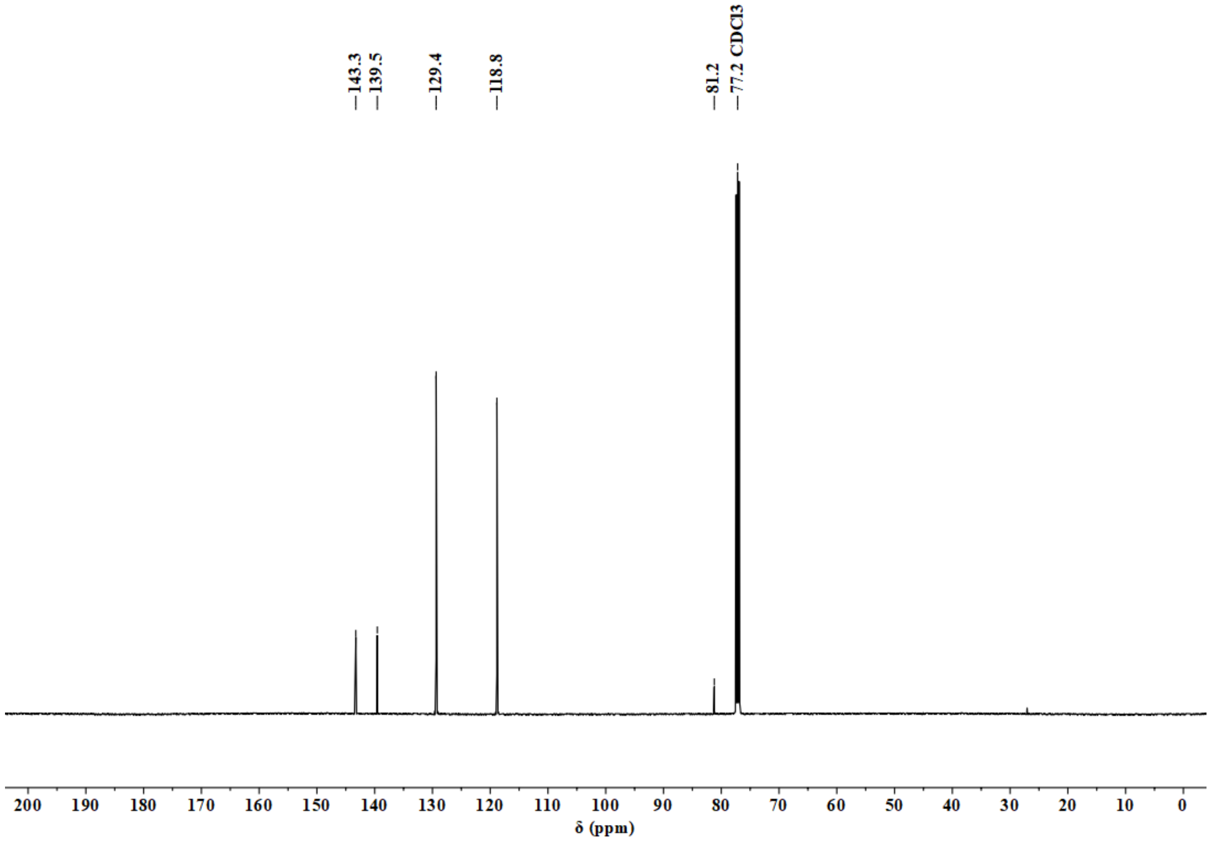

Figure S2. ^{13}C NMR –Tris(4-azidophenyl)methanol (**1**), 126 MHz, $CDCl_3$.

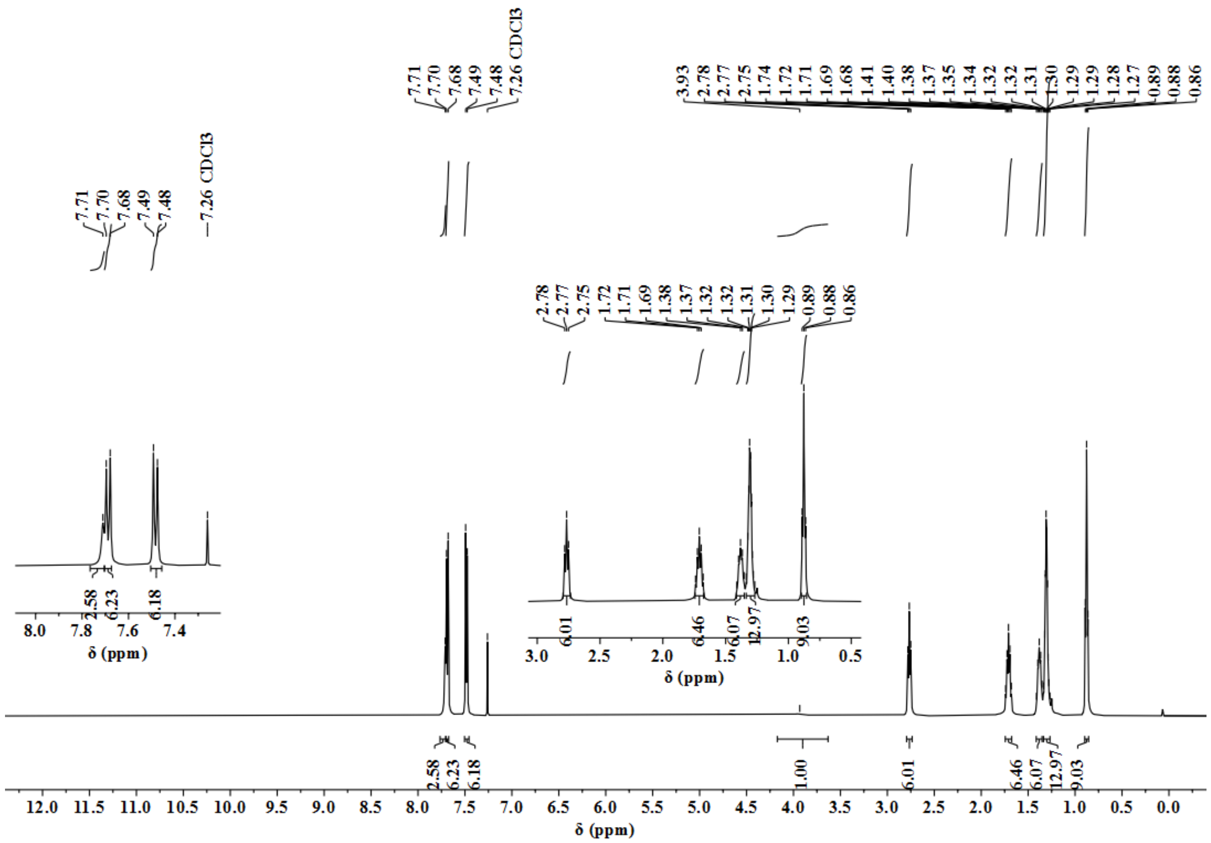

Figure S3. ^{1}H NMR – Tris(4-(4-hexyl-1H-1,2,3-triazol-1-yl)phenyl)methanol (**2a**), 500 MHz, $CDCl_3$.

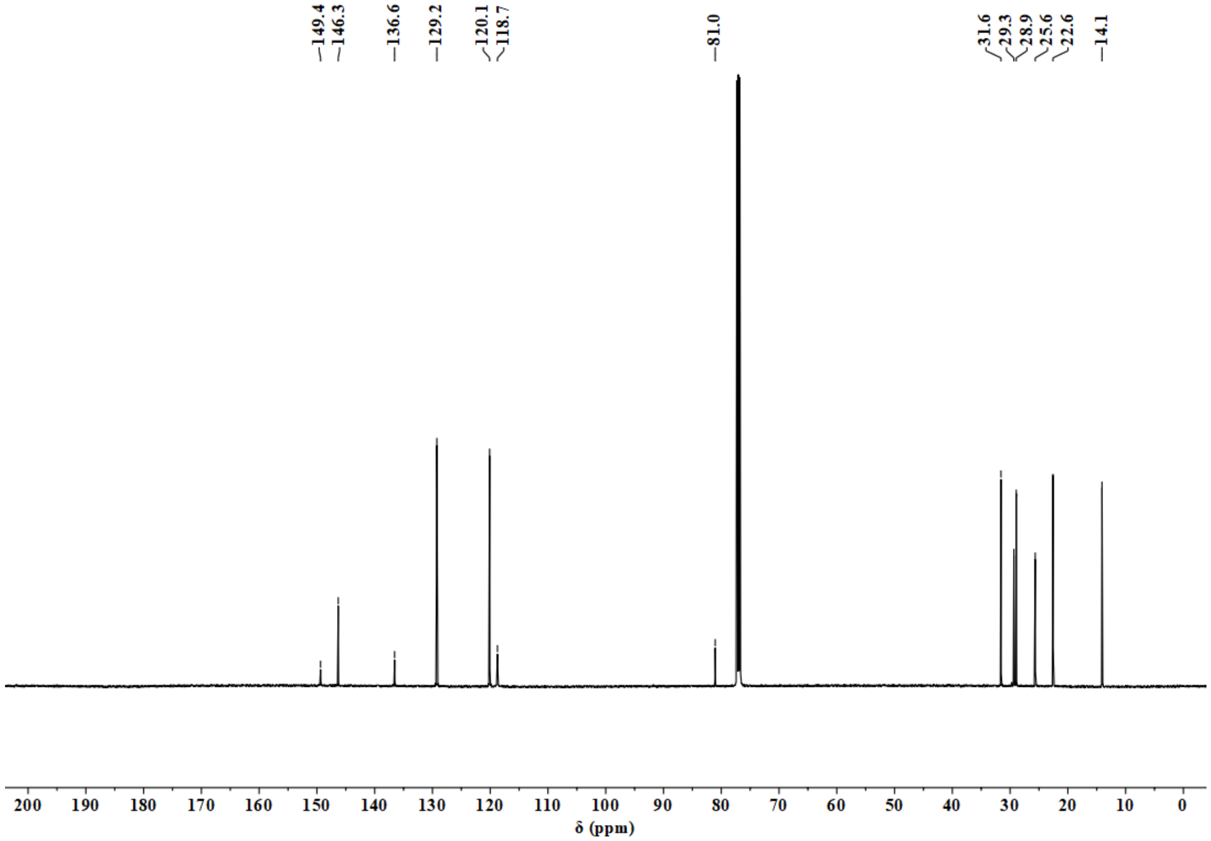

Figure S4. ^{13}C NMR –Tris(4-(4-hexyl-1H-1,2,3-triazol-1-yl)phenyl)methanol (**2a**), 126 MHz, $CDCl_3$.

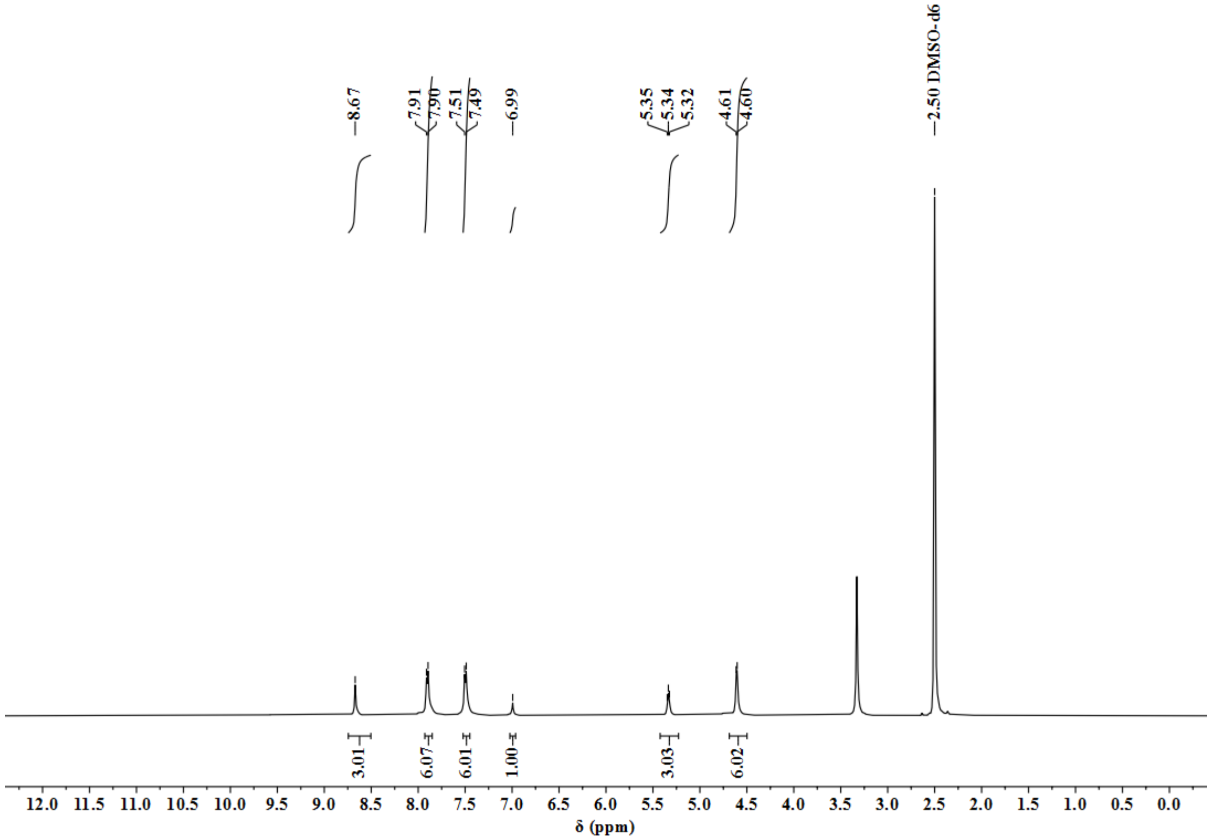

Figure S5. ^{1}H NMR –(((hydroxymethanetriyl)tris(benzene-4,1-diyl))tris(1H-1,2,3-triazole-1,4-diyl))trimethanol (**2b**), 500 MHz, DMSO-d_6.

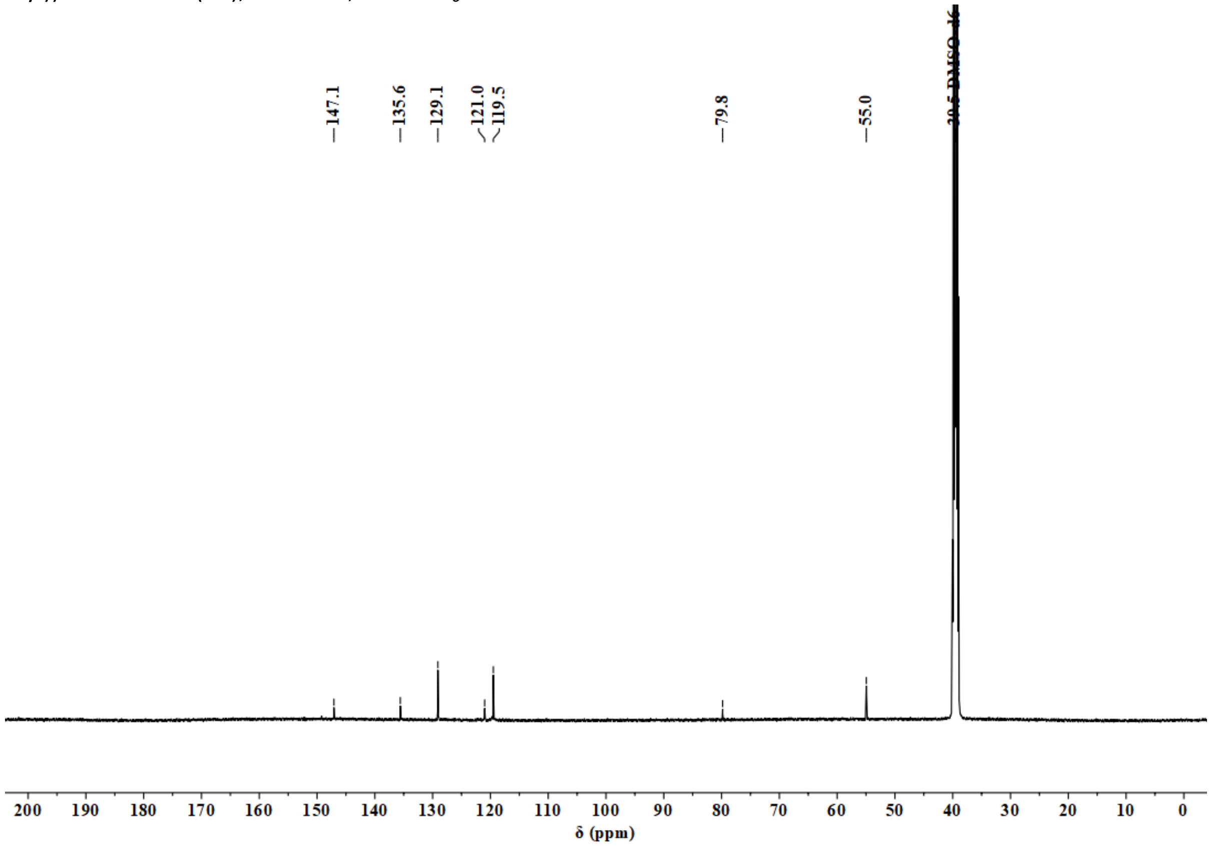

Figure S6. ^{13}C NMR – (((hydroxymethanetriyl)tris(benzene-4,1-diyl))tris(1H-1,2,3-triazole-1,4-diyl))trimethanol (**2b**), 126 MHz, DMSO-d_6.

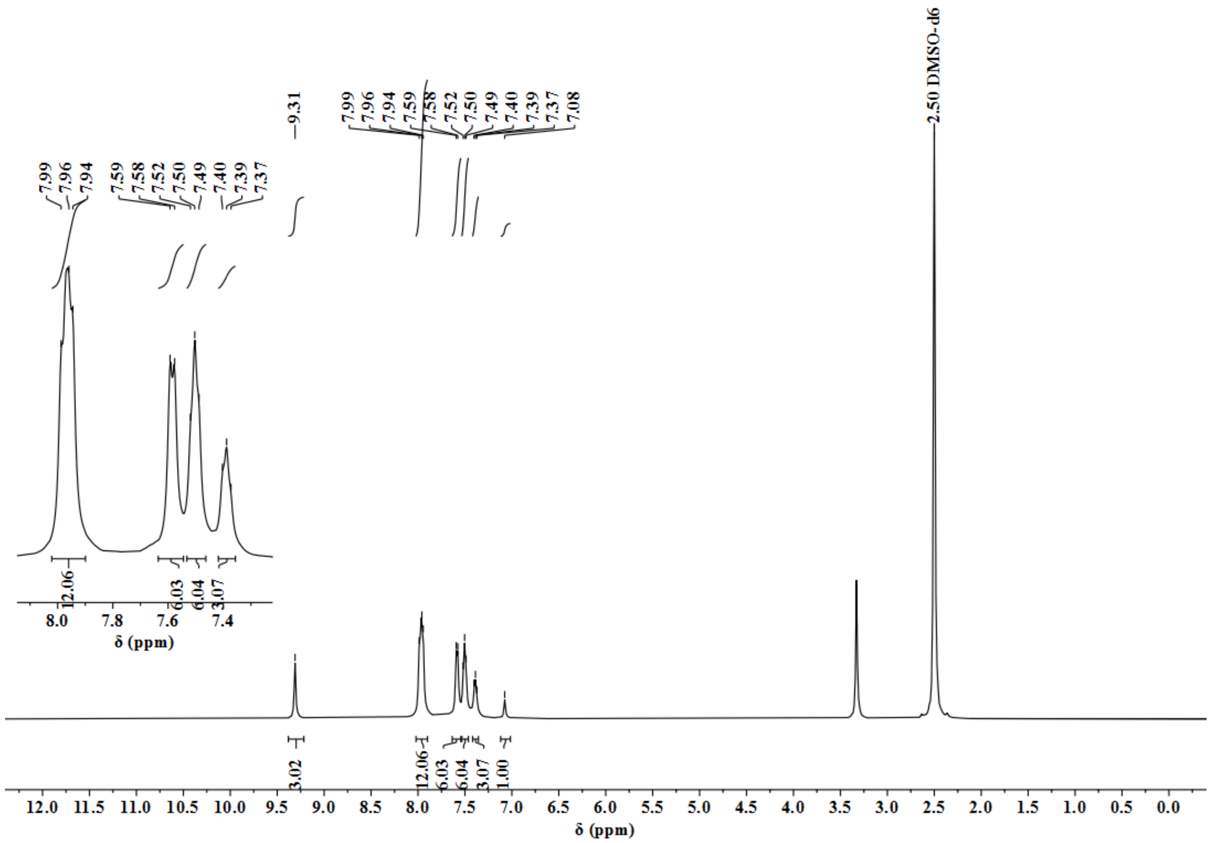

Figure S7. ^{1}H NMR – Tris(4-(4-phenyl-1H-1,2,3-triazol-1-yl)phenyl)methanol (**2c**), 500 MHz, DMSO-d_6.

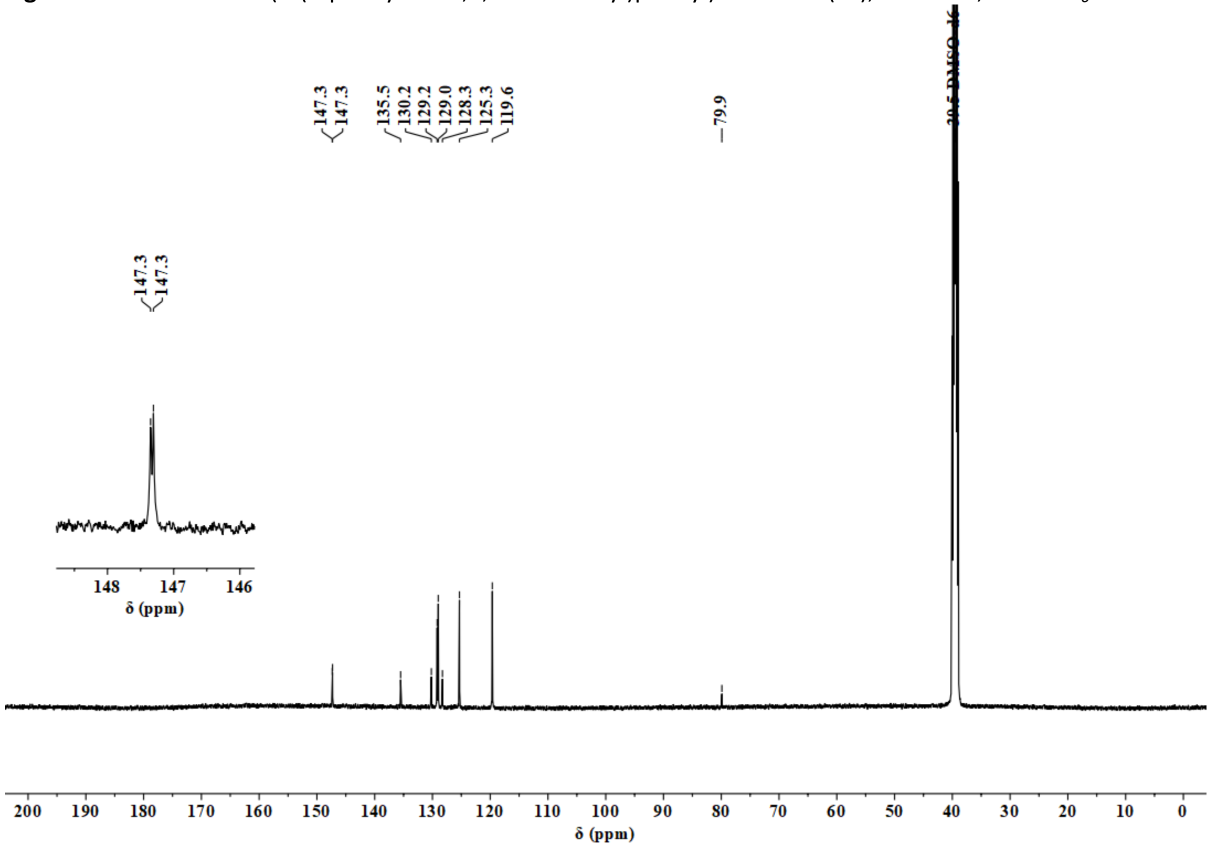

Figure S8. ^{13}C NMR – Tris(4-(4-phenyl-1H-1,2,3-triazol-1-yl)phenyl)methanol (**2c**), 126 MHz, DMSO-d_6.

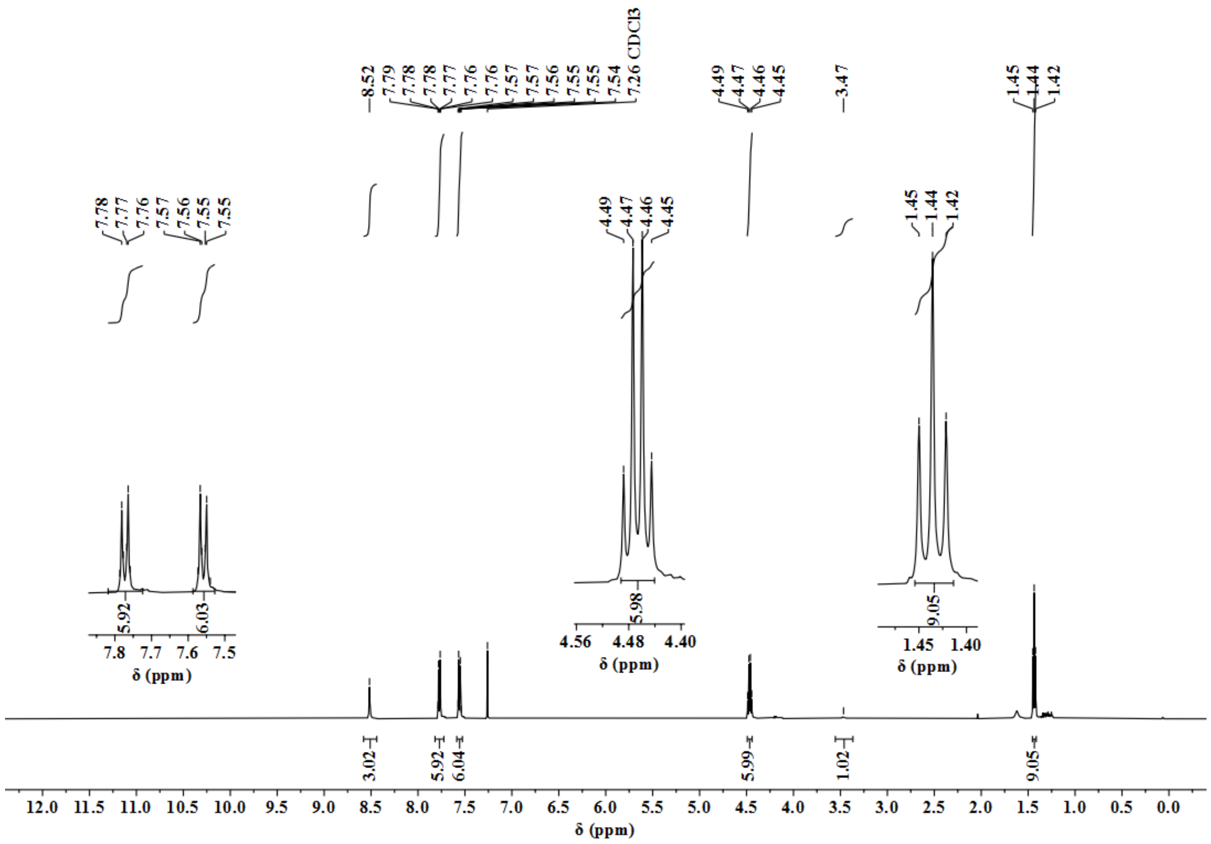

Figure S9. ^{1}H NMR –Triethyl 1,1',1''-((hydroxymethanetriyl)tris(benzene-4,1-diyl))tris(1H-1,2,3-triazole-4-carboxylate) (**2d**), 500 MHz, $CDCl_3$.

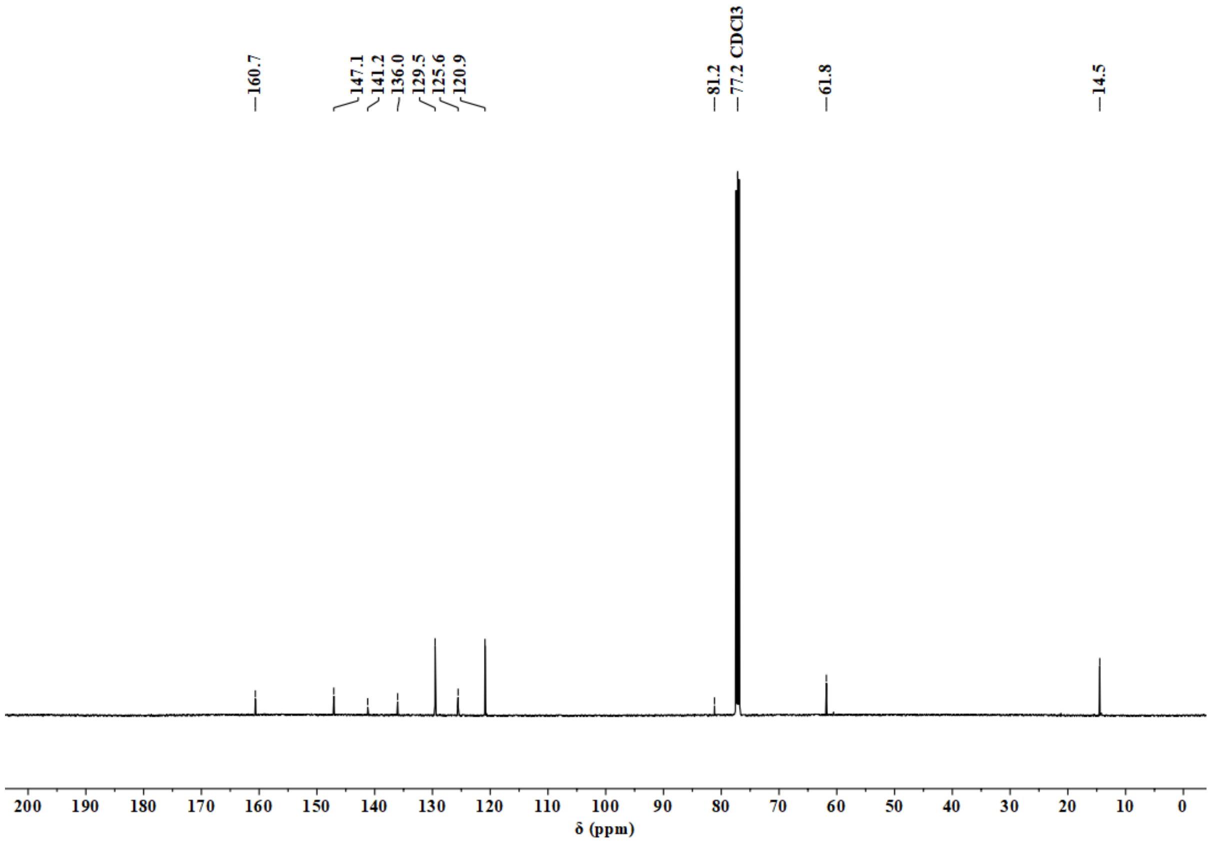

Figure S10. ^{13}C NMR – Triethyl 1,1',1''-((hydroxymethanetriyl)tris(benzene-4,1-diyl))tris(1H-1,2,3-triazole-4-carboxylate) (**2d**), 126 MHz, $CDCl_3$.

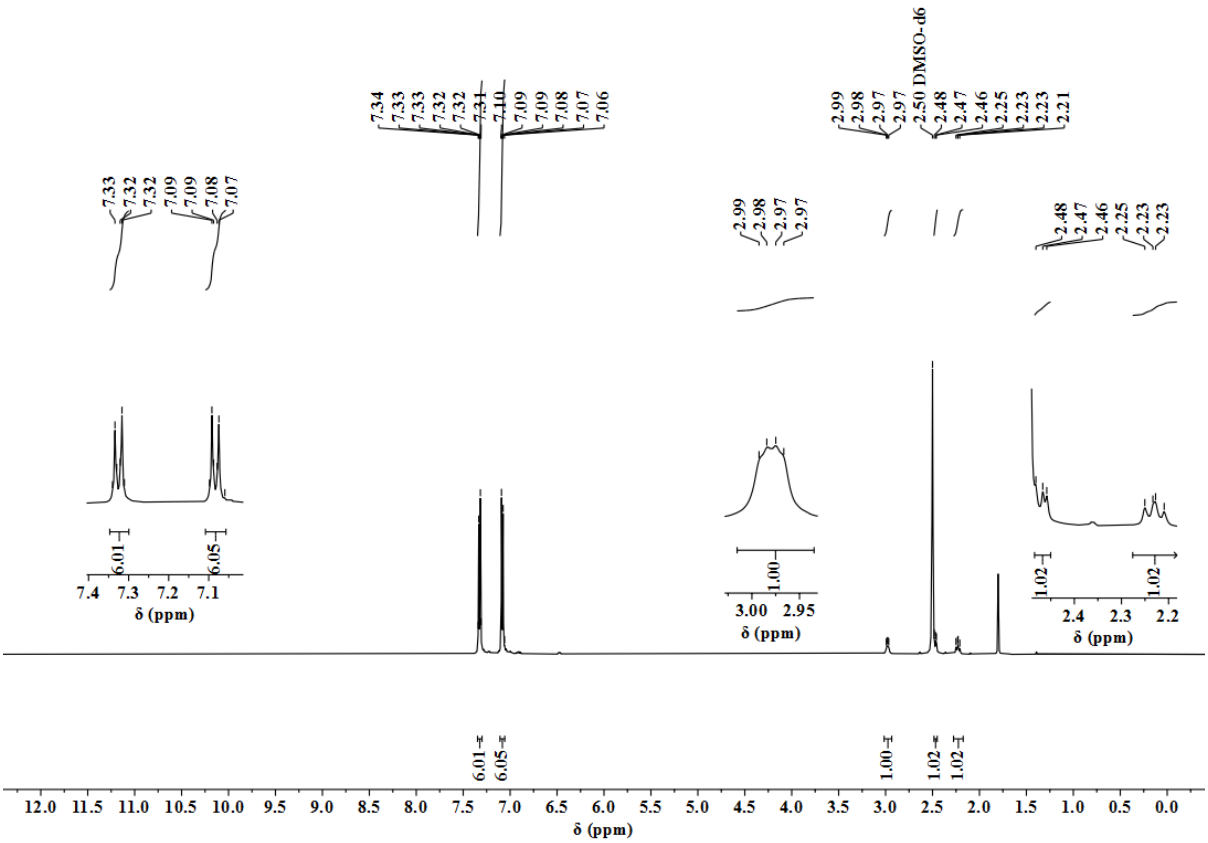

Figure S11. ^1H NMR – S-(tris(4-azidophenyl)methyl)-*L*-cysteine (**3 *L* -a**), 500 MHz, DMSO-d_6.

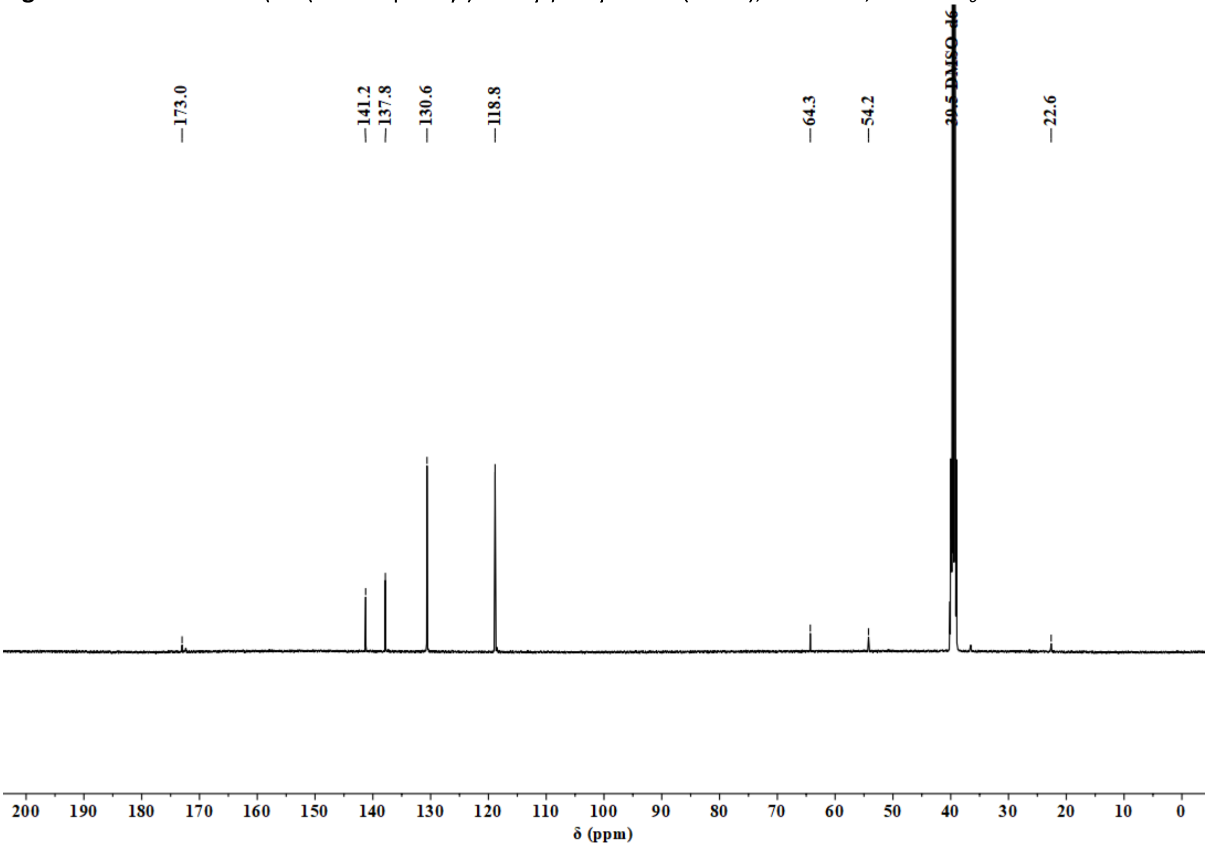

Figure S12. ^{13}C NMR – S-(tris(4-azidophenyl)methyl)-*L*-cysteine (**3 *L* -a**), 126 MHz, DMSO-d_6.

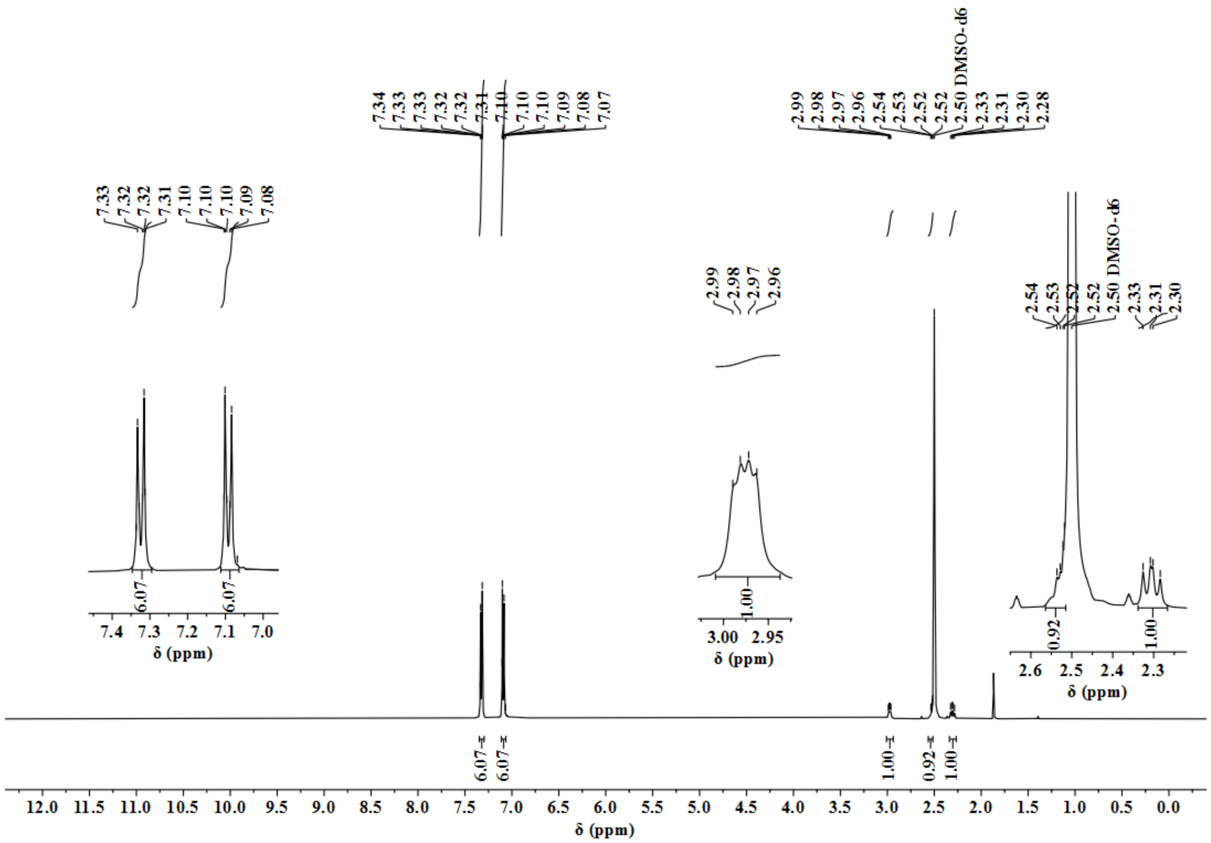

Figure S13. ^{1}H NMR – S-(tris(4-azidophenyl)methyl)-*D*-cysteine (**3 *D* -a**), 500 MHz, DMSO-d_6.

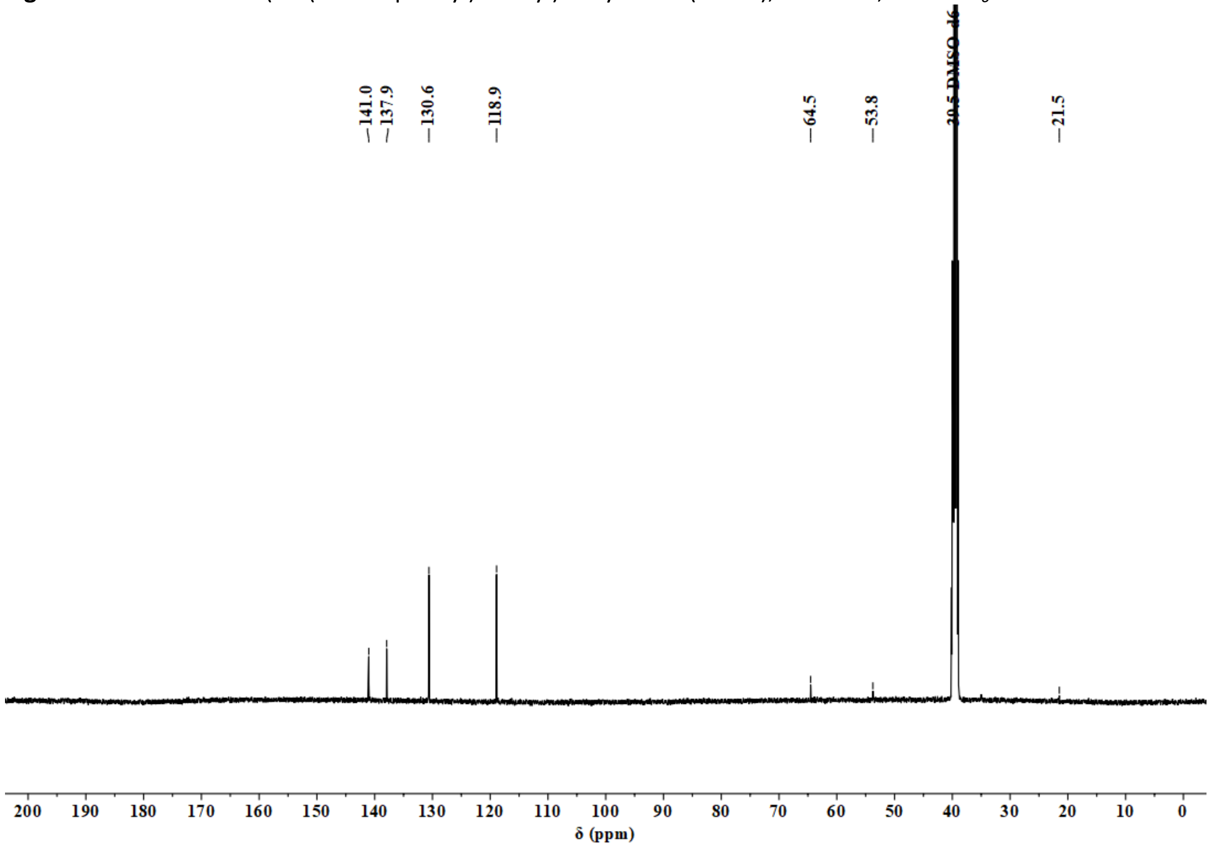

Figure S14. ^{13}C NMR – S-(tris(4-azidophenyl)methyl)-*D*-cysteine (**3 *D* -a**), 126 MHz, DMSO-d_6.

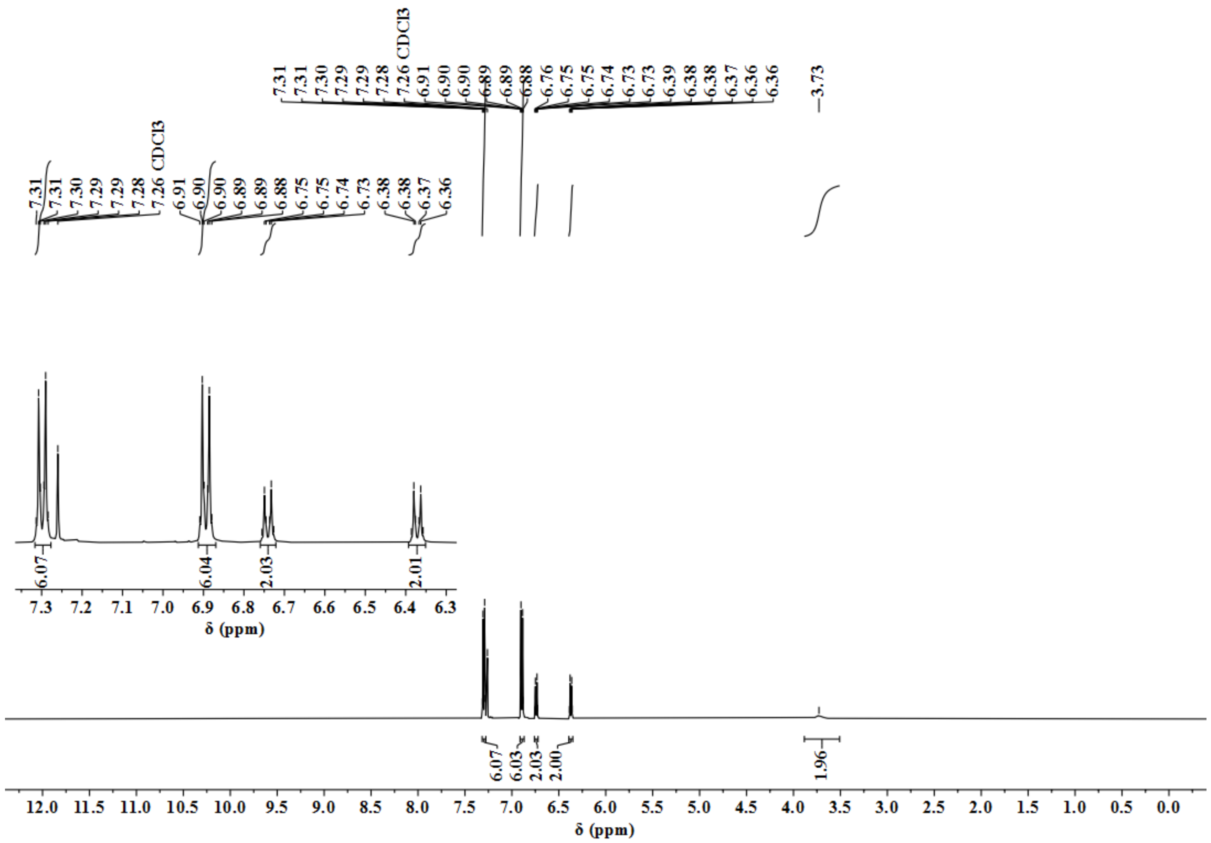

Figure S15. ^{1}H NMR – 4-((Tris(4-azidophenyl)methyl)thio)aniline (**3b**), 500 MHz, $CDCl_3$.

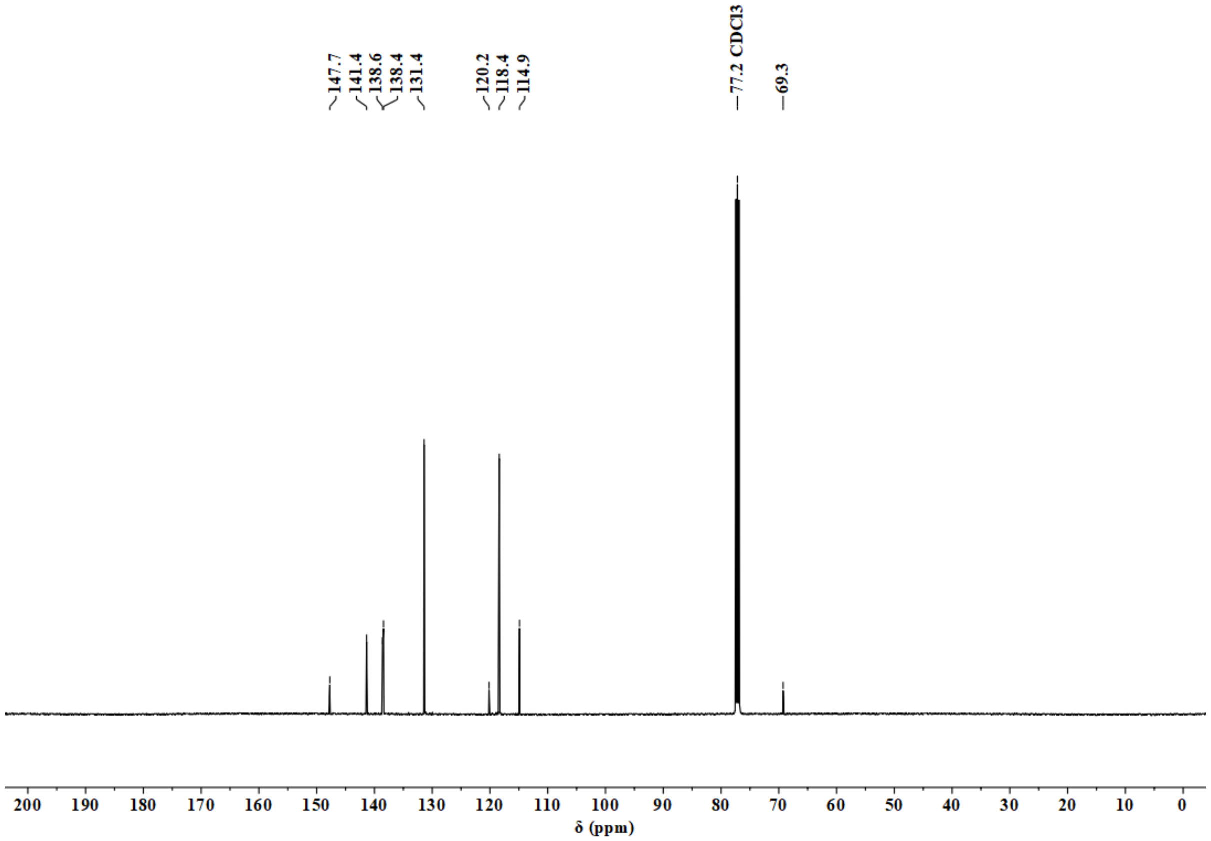

Figure S16. ^{13}C NMR – 4-((Tris(4-azidophenyl)methyl)thio)aniline (**3b**), 126 MHz, $CDCl_3$.

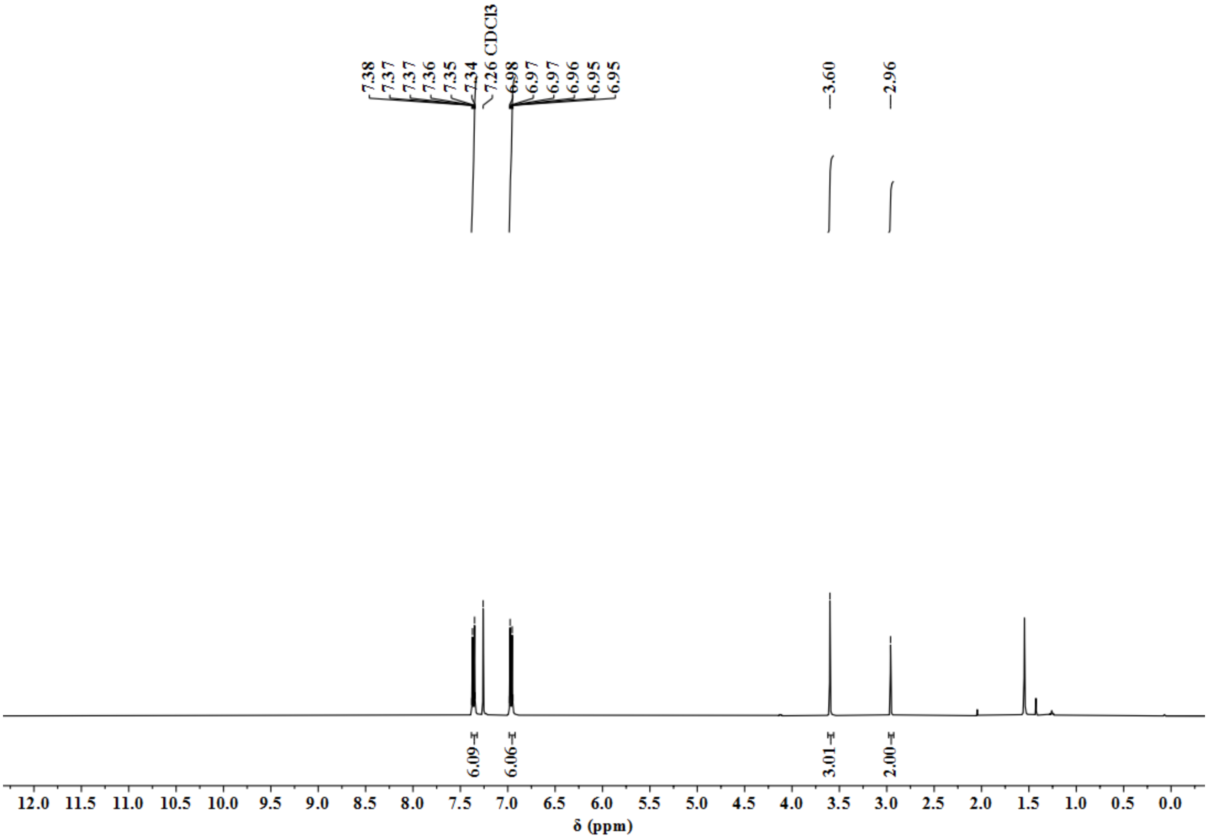

Figure S17. ^{1}H NMR – Methyl 2-((tris(4-azidophenyl)methyl)thio)acetate (**3c**), 400 MHz, $CDCl_3$.

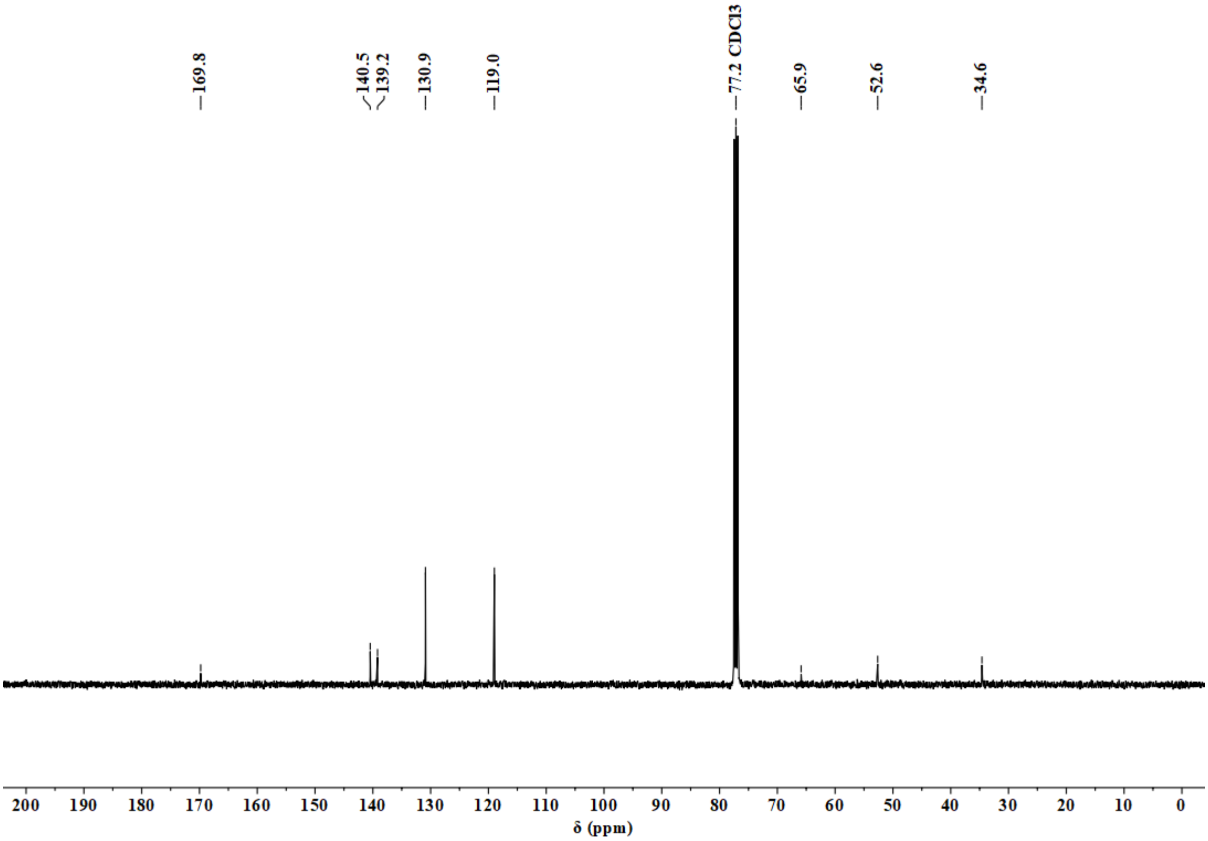

Figure S18. ^{13}C NMR – Methyl 2-((tris(4-azidophenyl)methyl)thio)acetate (**3c**), 101 MHz, $CDCl_3$.

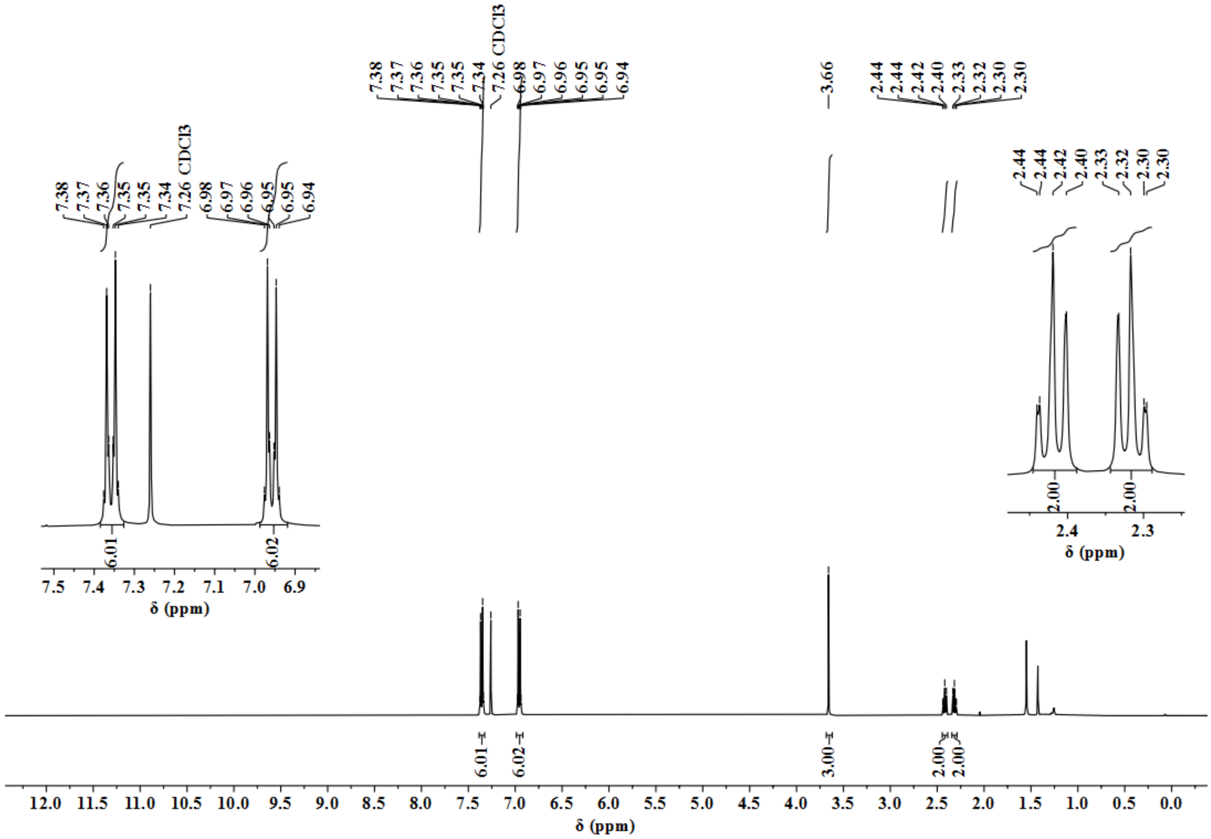

Figure S19. ^{1}H NMR – Methyl 3-((tris(4-azidophenyl)methyl)thio)propanoate (**3d**), 400 MHz, $CDCl_3$.

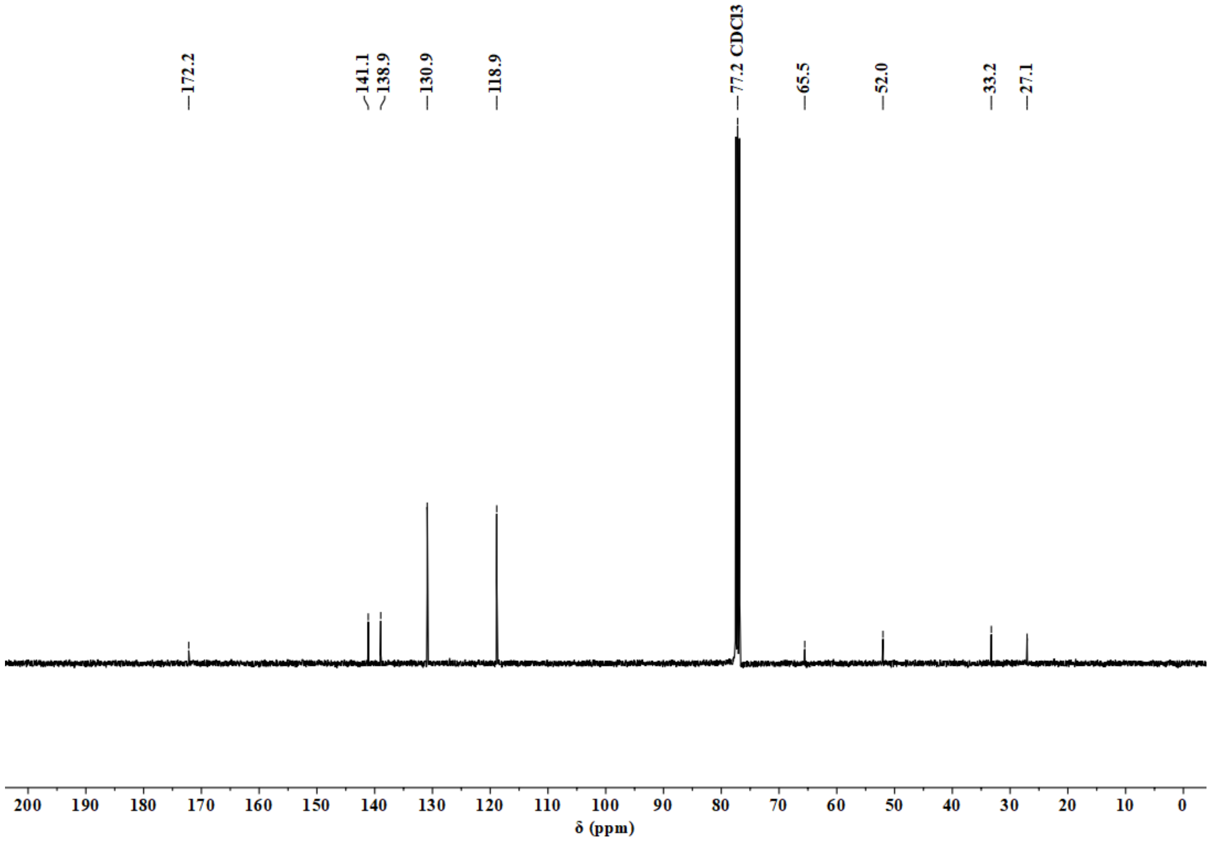

Figure S20. ^{13}C NMR – Methyl 3-((tris(4-azidophenyl)methyl)thio)propanoate (**3d**), 101 MHz, $CDCl_3$.

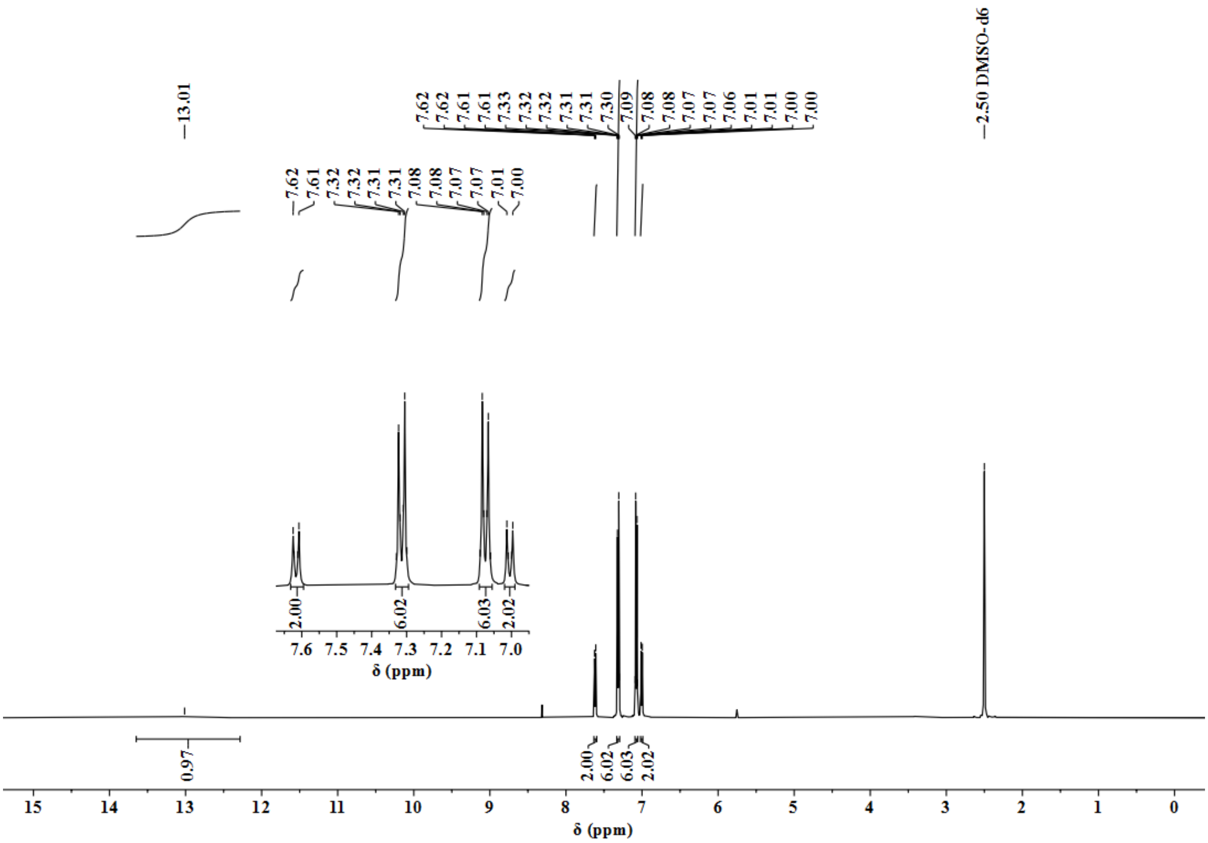

Figure S21. ^{1}H NMR – 4-((Tris(4-azidophenyl)methyl)thio)benzoic acid (**3e**), 500 MHz, DMSO-d_6.

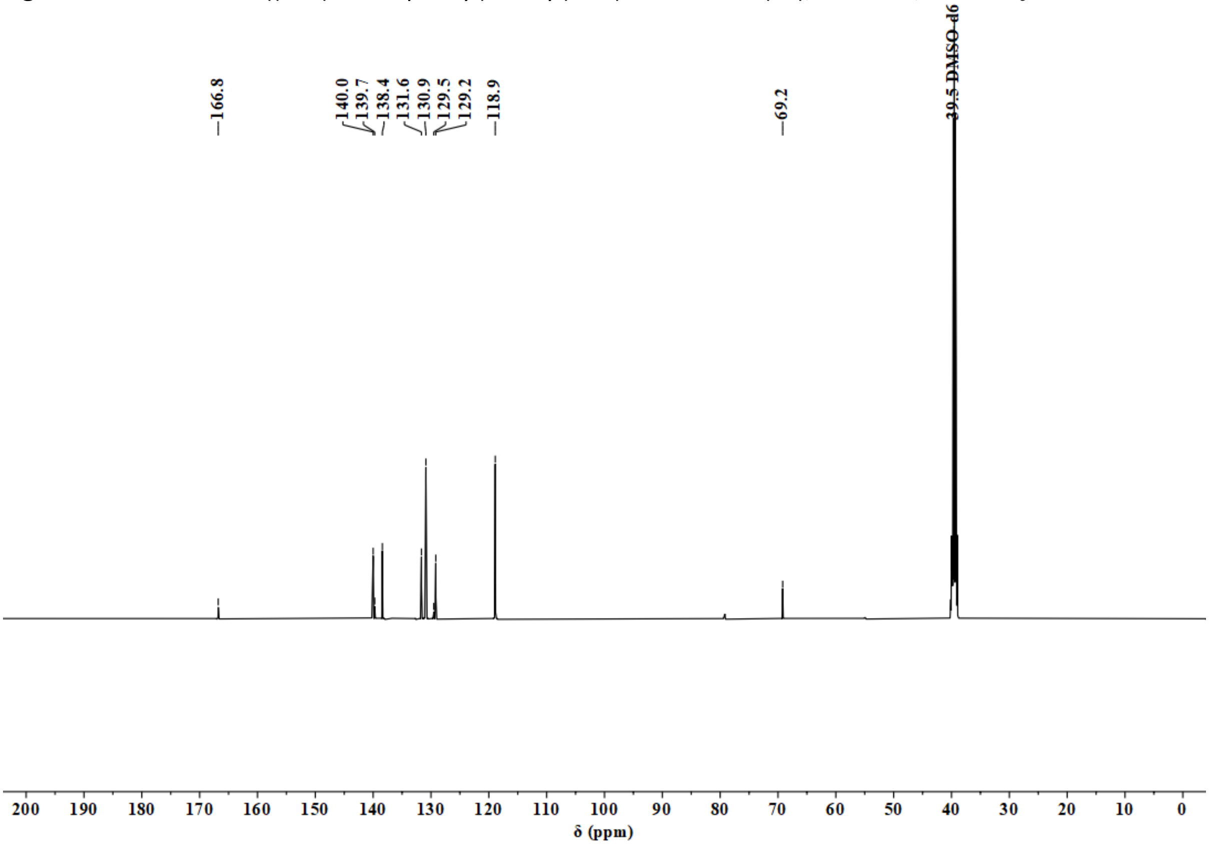

Figure S22. ^{13}C NMR – 4-((Tris(4-azidophenyl)methyl)thio)benzoic acid (**3e**), 126 MHz, DMSO-d_6.

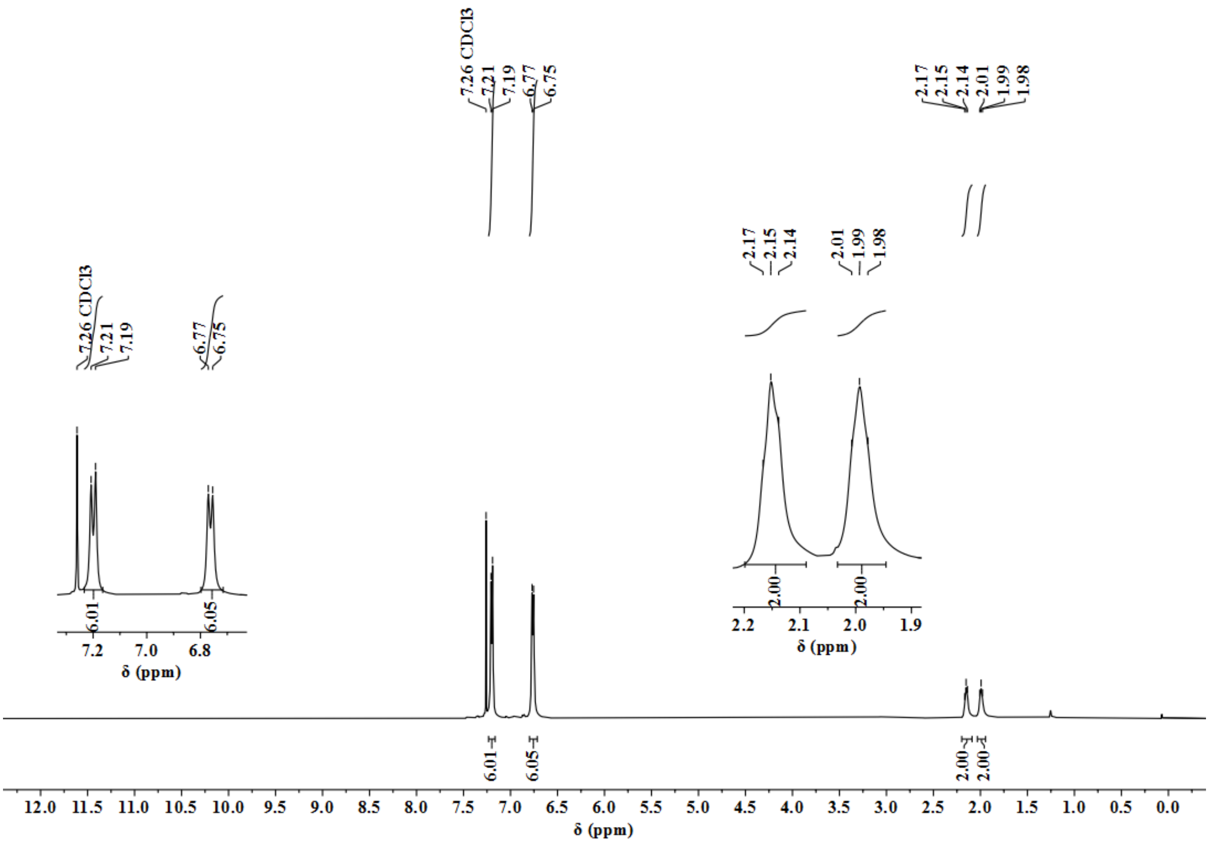

Figure S23. ^{1}H NMR – 3-((Tris(4-azidophenyl)methyl)thio)propanoic acid (**3f**), 500 MHz, $CDCl_3$.

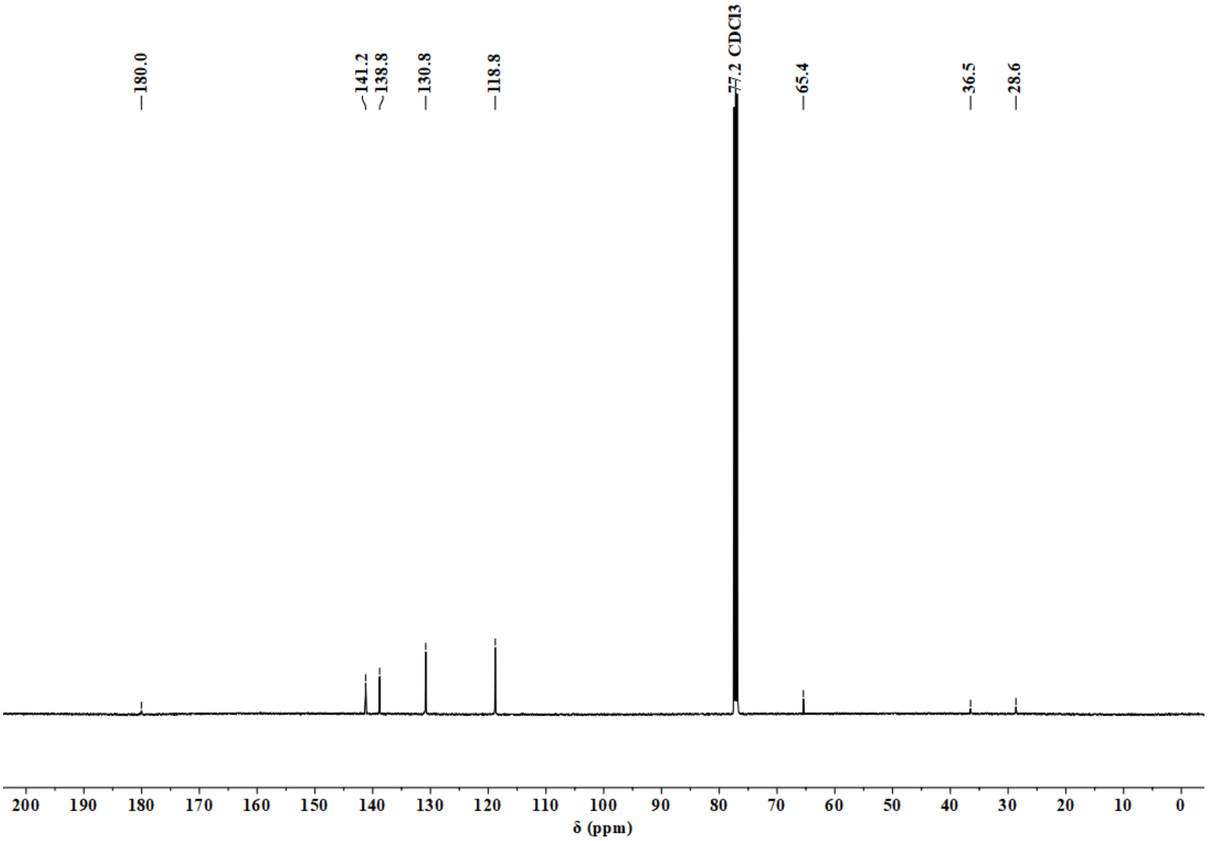

Figure S24. ^{13}C NMR – 3-((Tris(4-azidophenyl)methyl)thio)propanoic acid (**3f**), 126 MHz, $CDCl_3$.

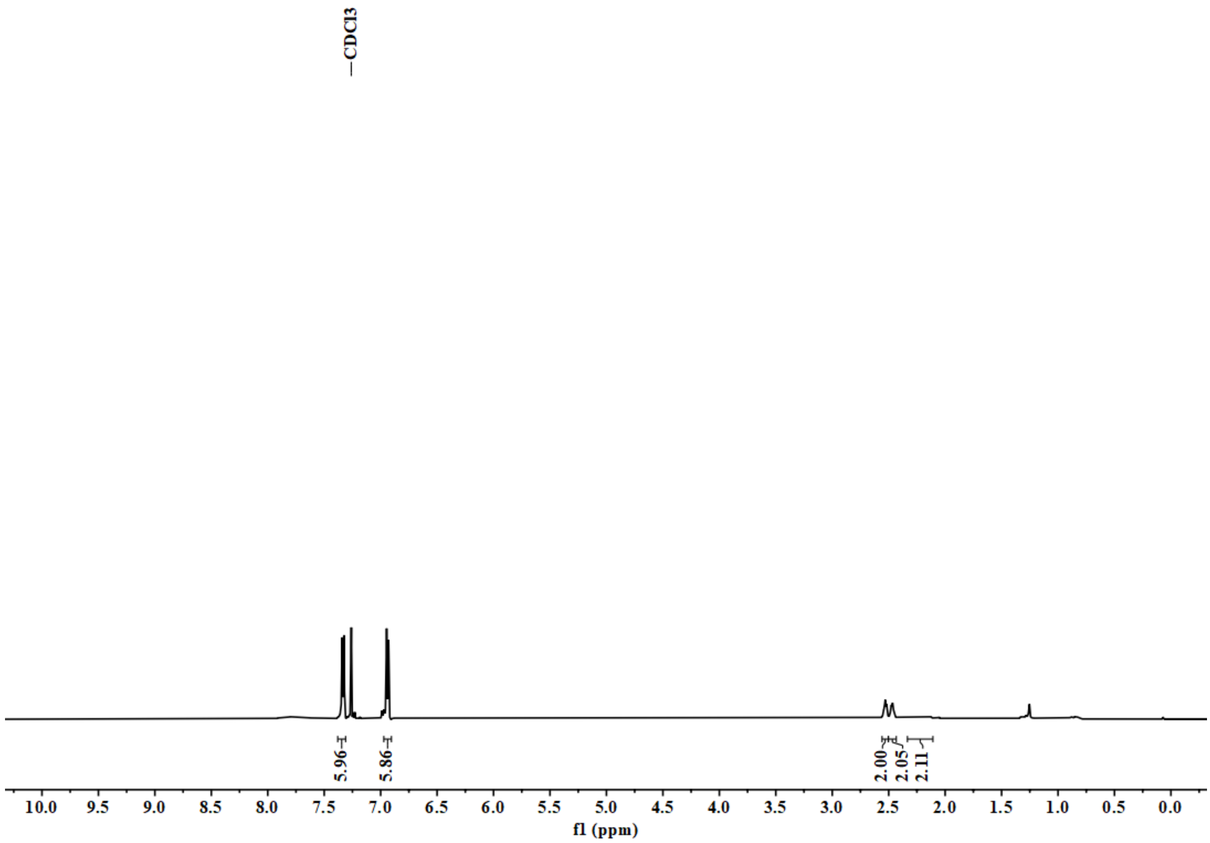

Figure S25. ^{1}H NMR – 2-((tris(4-azidophenyl)methyl)thio)ethan-1-amine (**3g**), 500 MHz, $CDCl_3$.

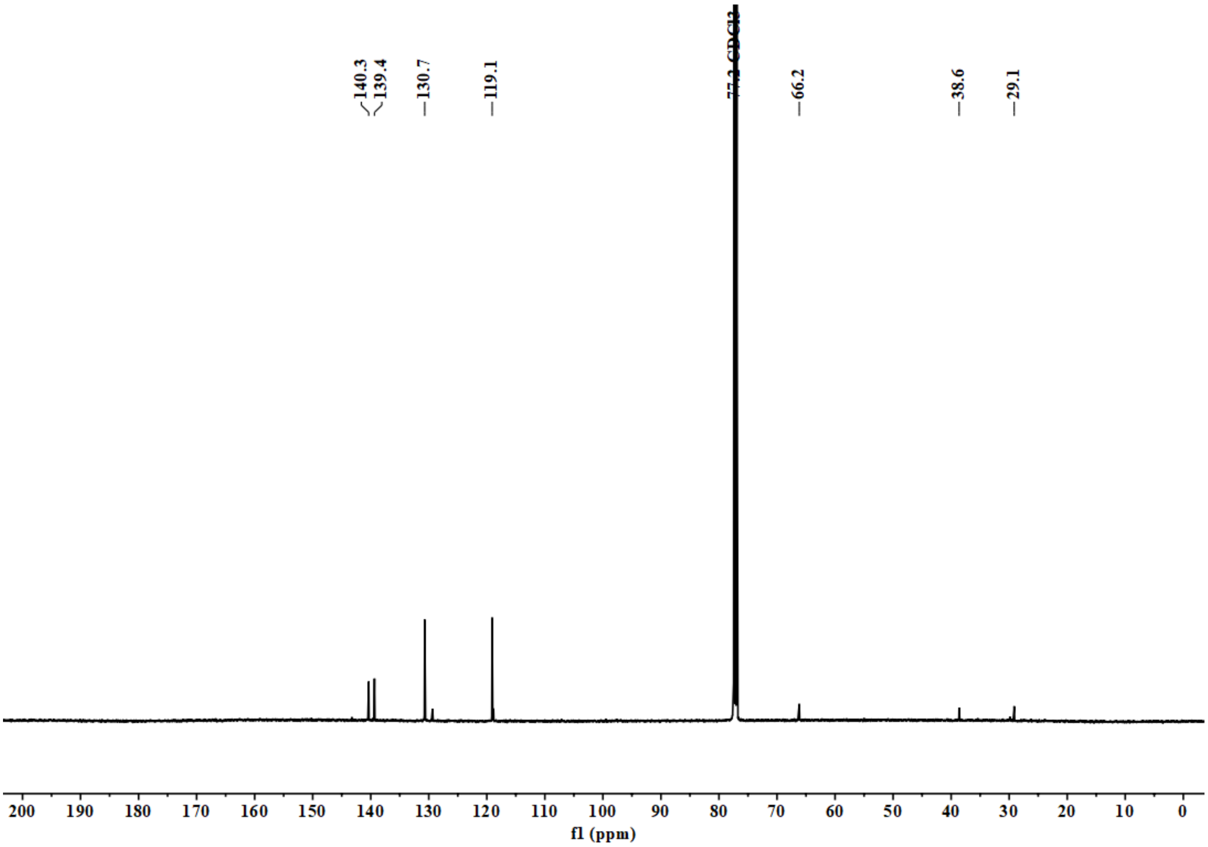

Figure S26. ^{13}C NMR – 2-((tris(4-azidophenyl)methyl)thio)ethan-1-amine (**3g**), 126 MHz, $CDCl_3$.

7. LC-MS and ESI-MS spectra

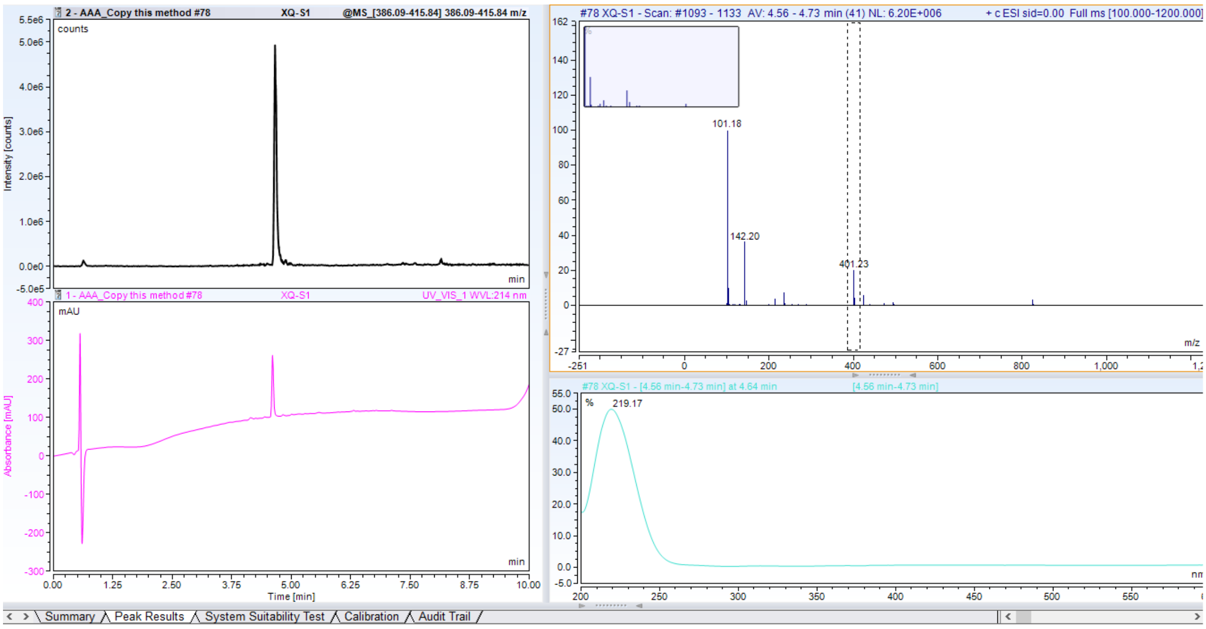

Figure S27. LC-MS spectra for peptoid **11a** $[C_{21}H_{24}N_2O_4S+H]^+$: Calc. *m/z* = 401.1457, found *m/z* =401.23

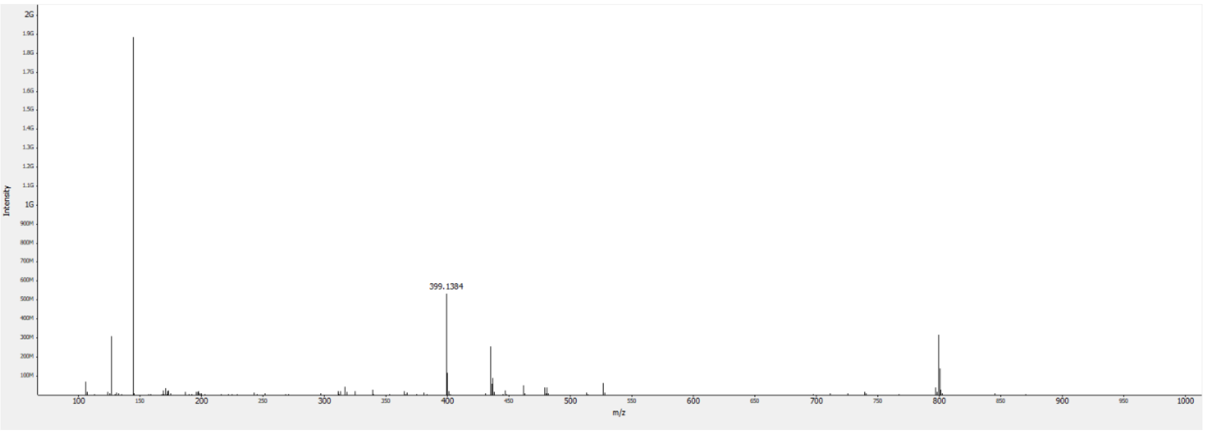

Figure S28. HRMS (ESI) spectra for peptoid **11a** $[C_{21}H_{24}N_2O_4S-H]^+$: Calc. *m/z* = 399.1457, found *m/z* =399.1384

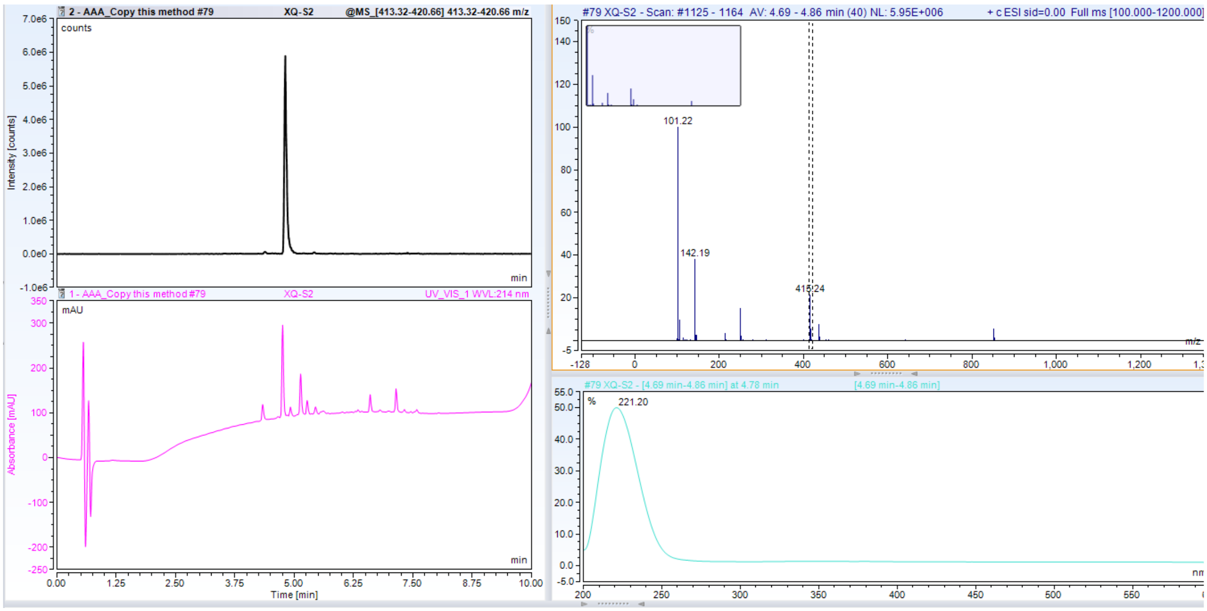

Figure S29. LC-MS spectra for peptoid **11b** [$C_{22}H_{26}N_2O_4S$+H]$^+$: Calc. *m/z* = 415.1613, found *m/z* =415.24

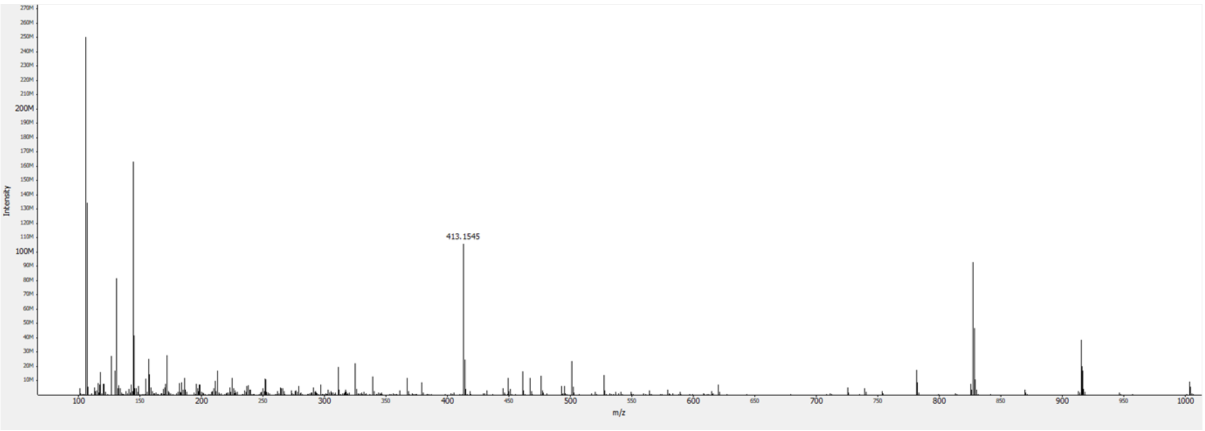

Figure S30. HRMS (ESI) spectra for peptoid **11b** [$C_{22}H_{26}N_2O_4S$-H]$^+$: Calc. *m/z* = 413.1613, found *m/z* =413.1545

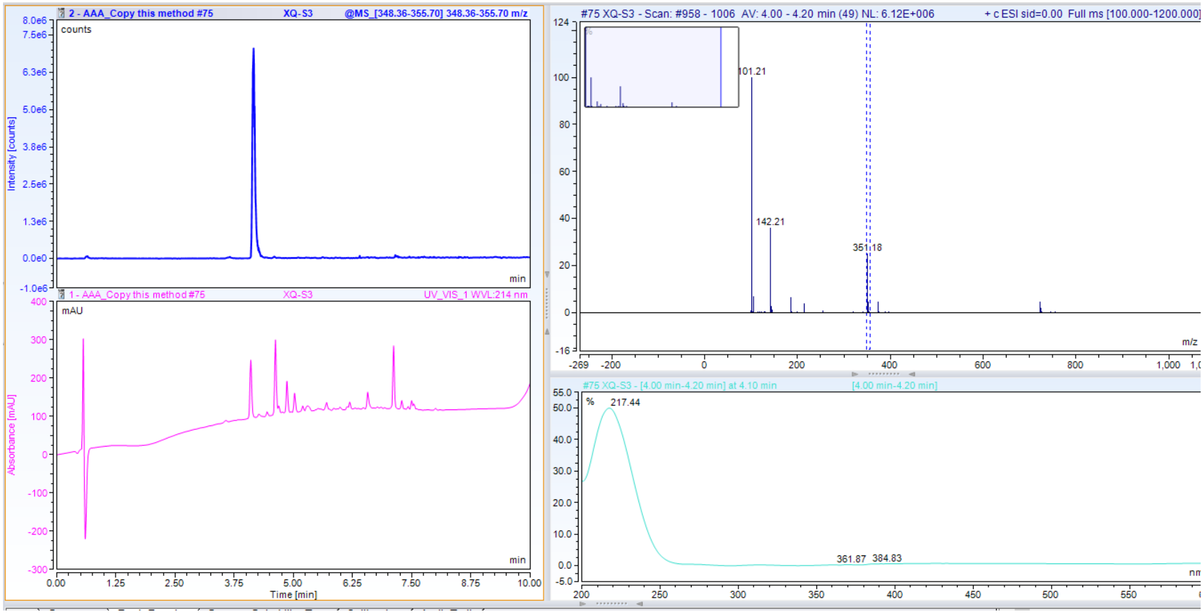

Figure S31. LC-MS spectra for peptoid **11c** [$C_{17}H_{22}N_2O_4S+H]^+$: Calc. *m/z* = 351.13, found *m/z* =351.18

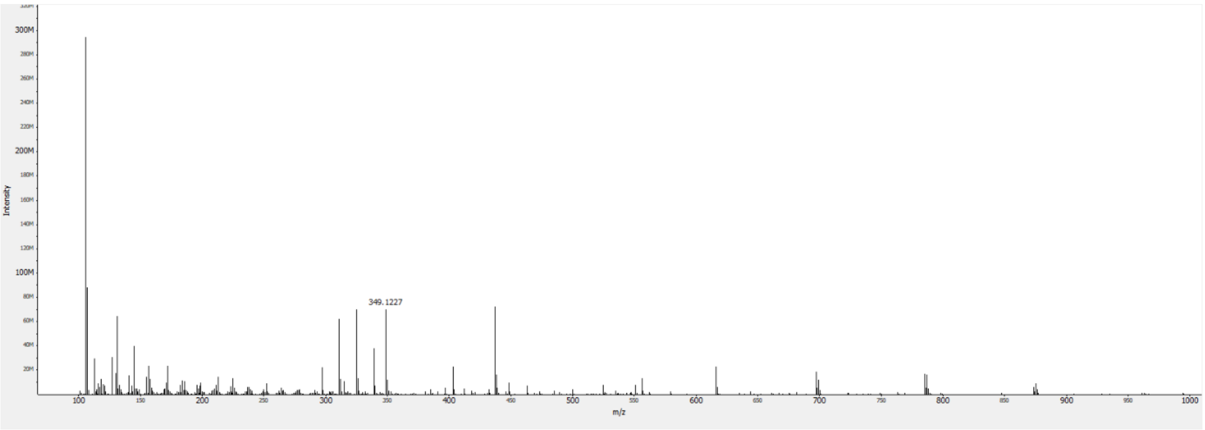

Figure S32. HRMS (ESI) spectra for peptoid **11c** [$C_{17}H_{22}N_2O_4S-H]^+$: Calc. *m/z* = 349.13, found *m/z* =349.1227

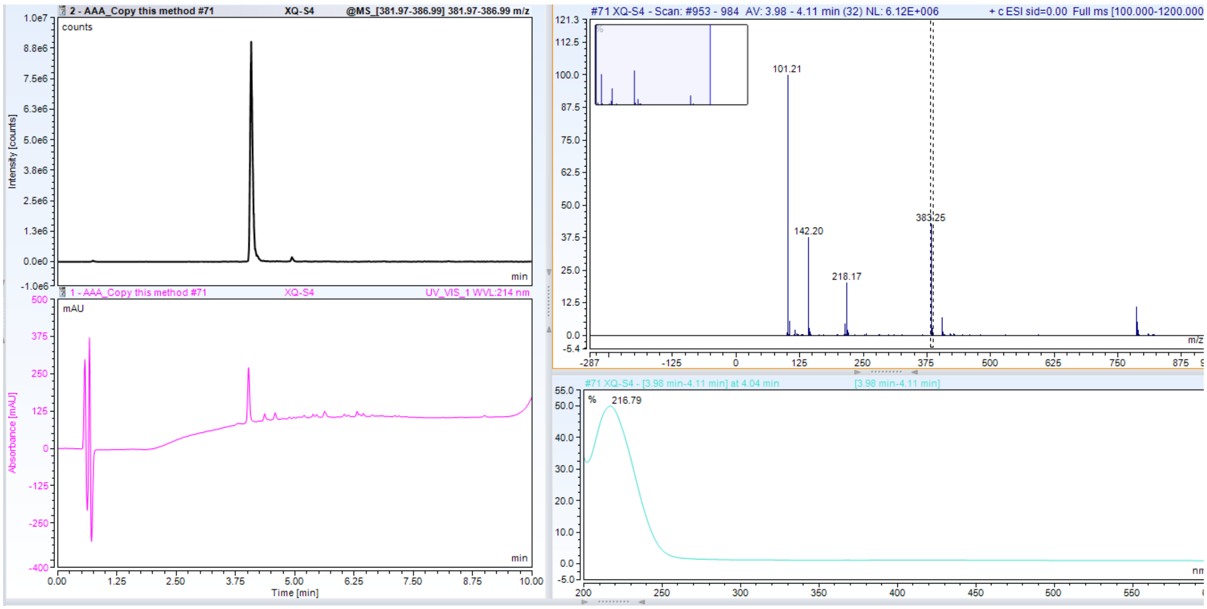

Figure S33. LC-MS spectra for peptoid **11d** $[C_{18}H_{26}N_2O_5S+H]^+$: Calc. m/z = 383.1564, found m/z =383.25

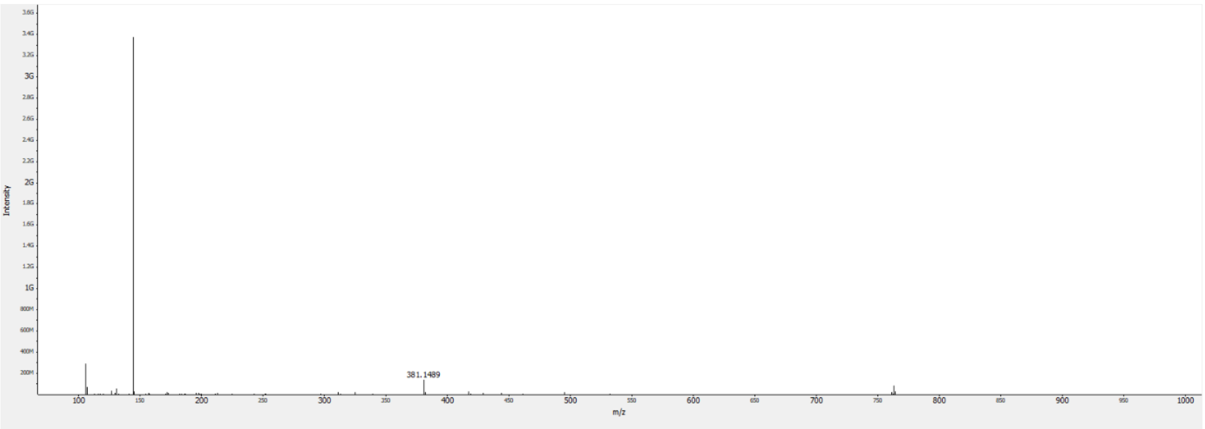

Figure S34. HRMS (ESI) spectra for peptoid **11d** $[C_{18}H_{26}N_2O_5S\ -H]^+$: Calc. m/z = 381.13, found m/z =381.1489

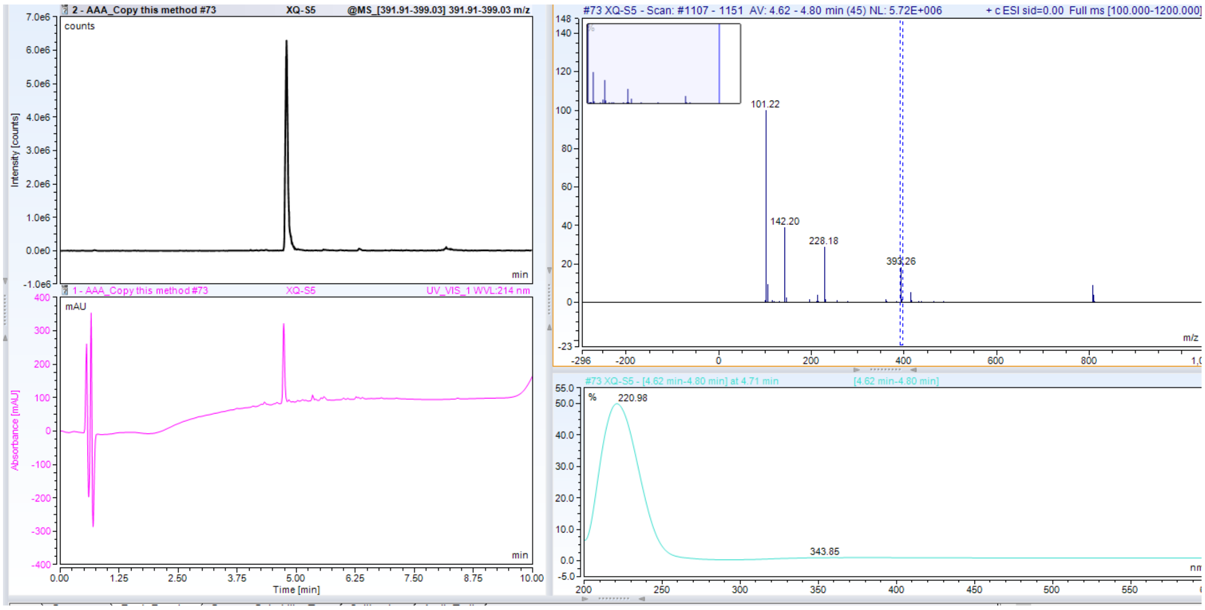

Figure S35. LC-MS spectra for peptoid **11e** $[C_{20}H_{28}N_2O_4S+H]^+$: Calc. *m/z* = 393.1770, found *m/z* =393.26

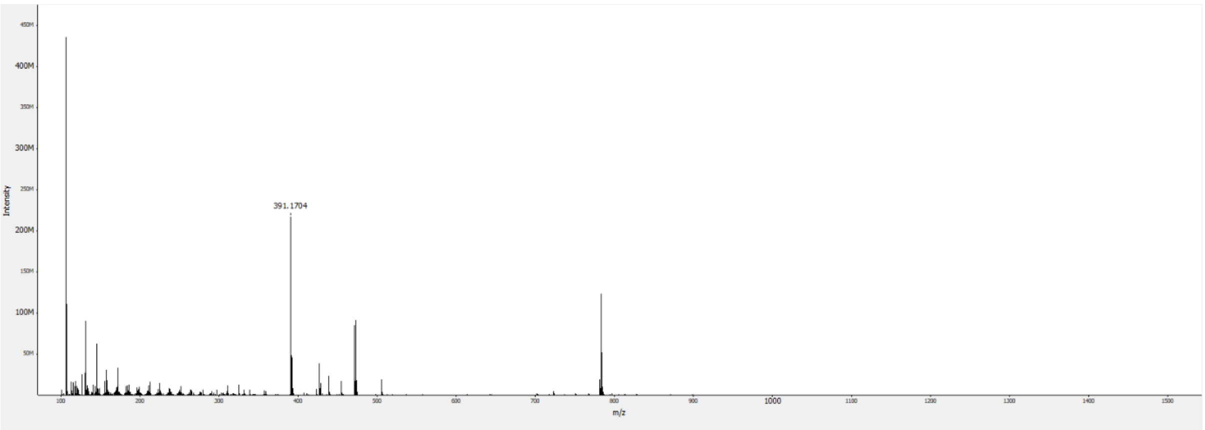

Figure S36. HRMS (ESI) spectra for peptoid **11e** $[C_{20}H_{28}N_2O_4S\ {-H}]^+$: Calc. *m/z* = 391.1770, found *m/z* =391.1704

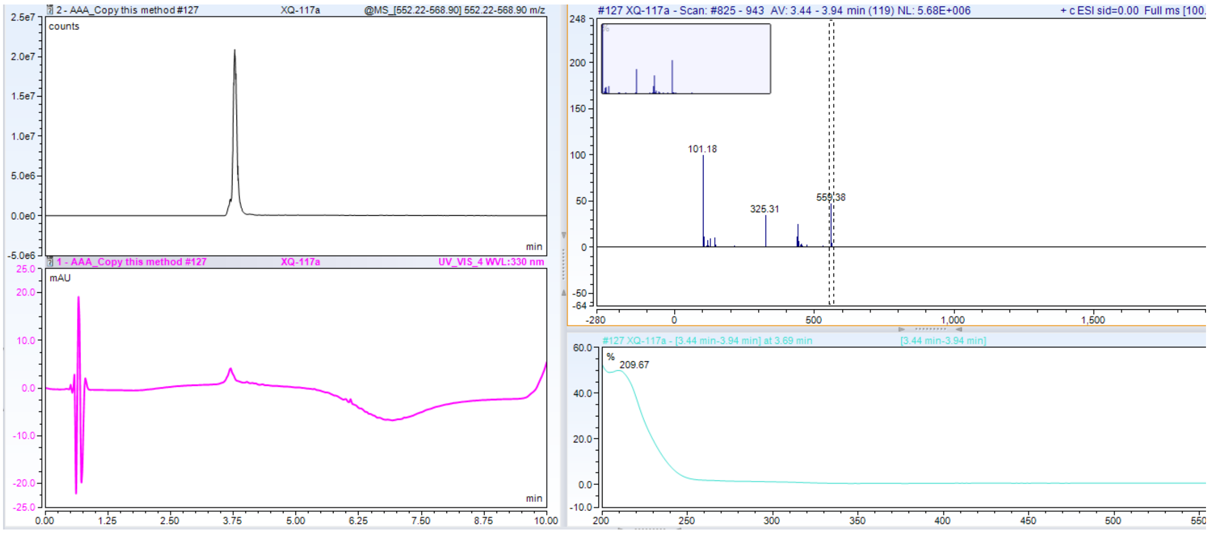

Figure S37. LC-MS spectra for peptoid **16a** $[C_{28}H_{38}N_4O_6S\ {+H}]^+$: Calc. *m/z* = 559.2512, found *m/z* =559.38

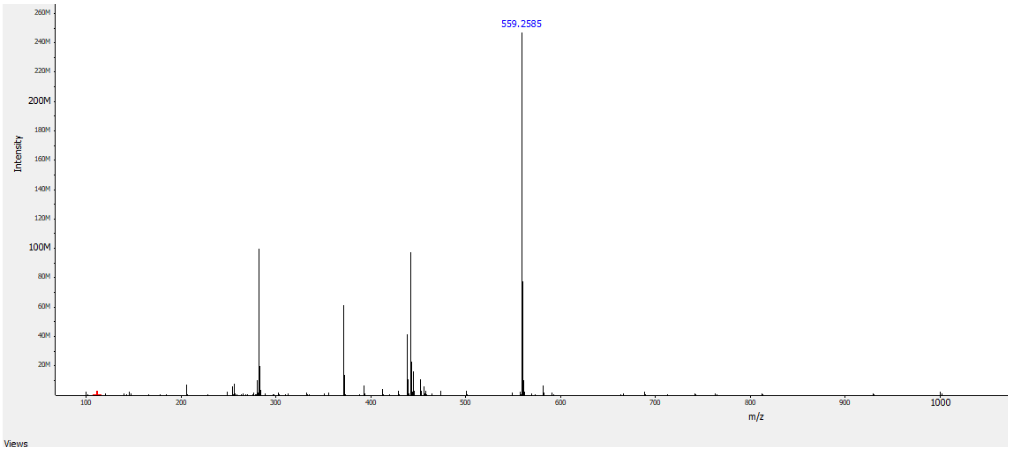

Figure S38. HRMS (ESI) spectra for peptoid **16a** $[C_{28}H_{38}N_4O_6S + H]^+$: Calc. *m/z* = 559.2512, found *m/z* =559.2585

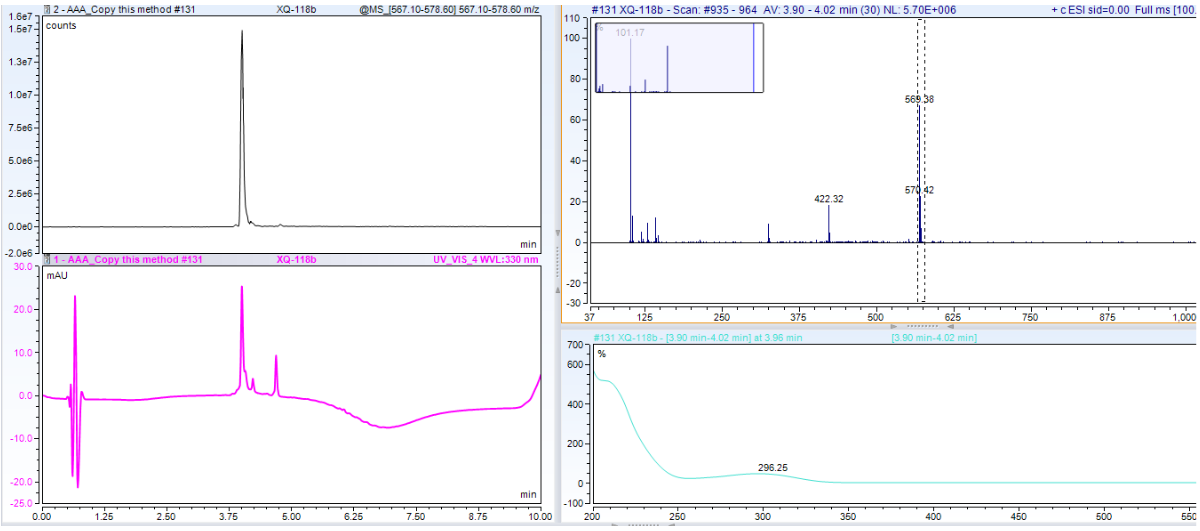

Figure S39. LC-MS spectra for peptoid **16b** $[C_{30}H_{40}N_4O_5S + H]^+$: Calc. *m/z* = 569.2719, found *m/z* =569.38

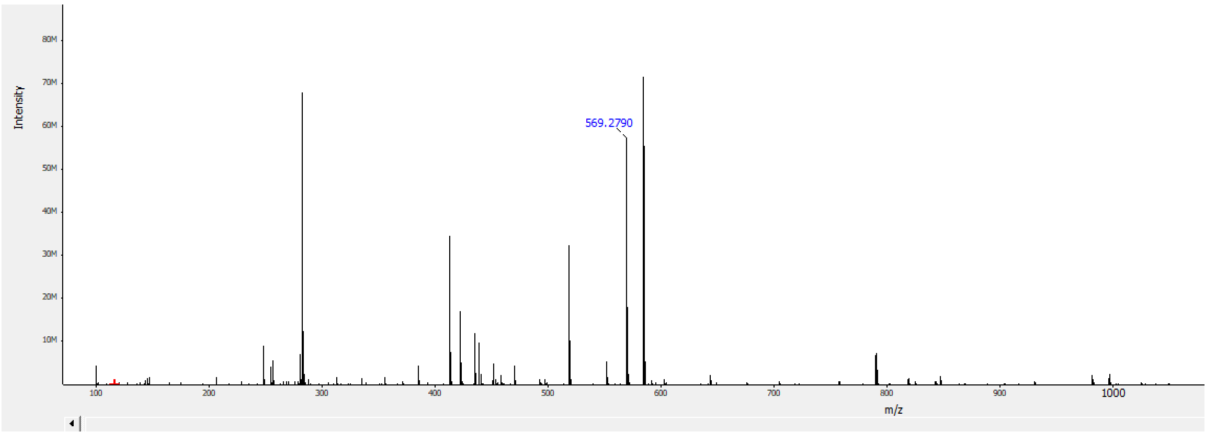

Figure S40. HRMS (ESI) spectra for peptoid **16b** $[C_{30}H_{40}N_4O_5S + H]^+$: Calc. *m/z* = 569.2719, found *m/z* =569.2790

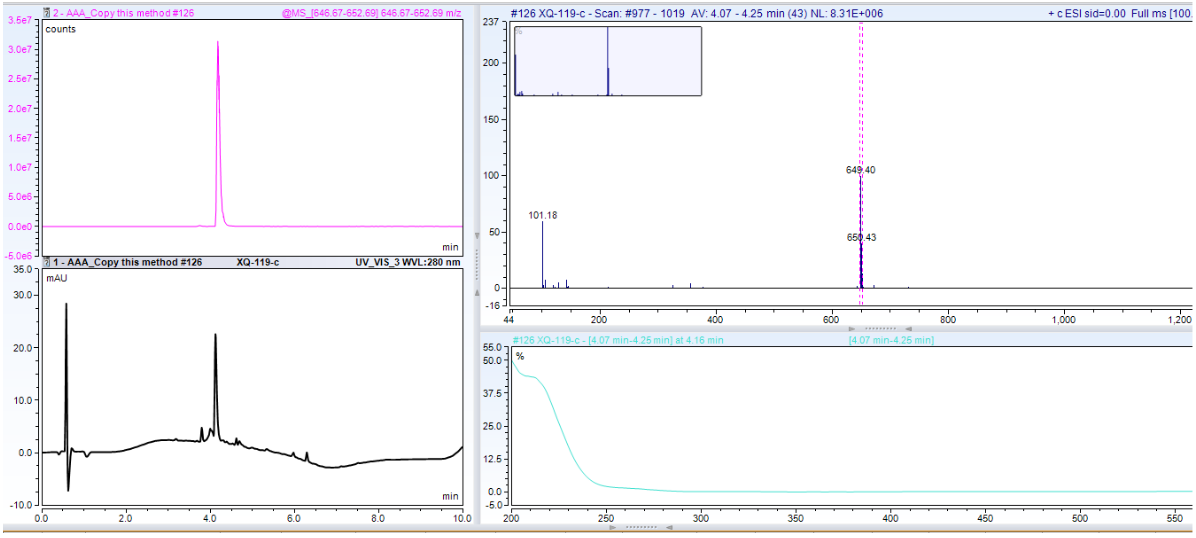

Figure S41. LC-MS spectra for peptoid **16c** $[C_{35}H_{44}N_4O_6S +H]^+$: Calc. *m/z* = 649.2982, found *m/z* =649.40

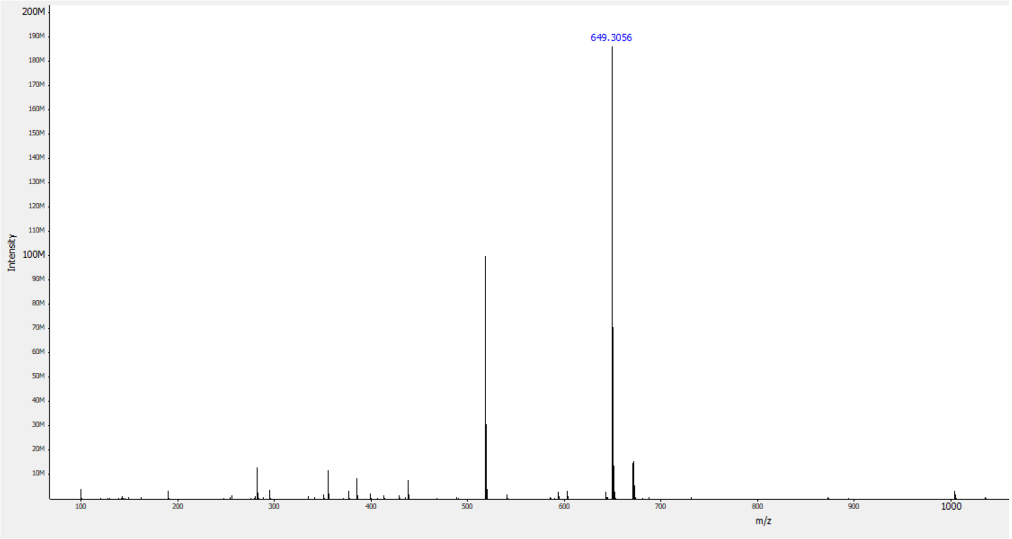

Figure S42. HRMS (ESI) spectra for peptoid **16c** $[C_{35}H_{44}N_4O_6S +H]^+$: Calc. *m/z* = 649.2982, found *m/z* =649.3056

8. X-Ray Diffractometry

The single-crystal X-ray diffraction study of **1** was carried out on a Bruker D8 Venture diffractometer with a PhotonII detector at 298(2) K using Mo-Kα radiation (λ = 0.71073 Å). Dual space methods (SHELXT) [G. M. Sheldrick, *Acta Crystallogr.* 2015, **A71**, 3-8] were used for structure solution, and refinement was carried out using SHELXL-2014 (full-matrix least-squares on F^2) [G. M. Sheldrick, *Acta Crystallogr.* 2015, **C71**, 3-8]. Hydrogen atoms were localized by difference electron density determination and refined using a riding model (H(O) free). A semi-empirical absorption correction was applied. The absolute structure could not be determined reliably (Parsons' x-parameter 0.2(10) [S. Parson, H. D. Flack, T. Wagner, *Acta Crystallogr.* 2013, **B69**, 249-259]

1: colourless crystals, $C_{19}H_{13}N_9O$, M_r = 383.38, crystal size 0.20 × 0.15 × 0.10 mm, orthorhombic, space

group $Pna2_1$ (No. 33), a = 10.422(3) Å, b = 17.540(6) Å, c = 10.109(3) Å, V = 1847.9(10) Å^3, Z = 4, ρ = 1.378 Mg/m^{-3}, μ(Mo-K$_\alpha$) = 0.09 mm^{-1}, F(000) = 792, T = 298 K, $2\theta_{max}$ = 55.0°, 38376 reflections, of which 4217 were independent (R_{int} = 0.065), 265 parameters, 2 restraints, R_1 = 0.038 (for 3742 I > 2σ(I)), wR_2 = 0.098 (all data), S = 1.03, largest diff. peak / hole = 0.12 / -0.15 e Å^{-3}, x = 0.2(10).

CCDC 2158276 (**1**) contains the supplementary crystallographic data for this paper. These data can be obtained free of charge from The Cambridge Crystallographic Data Centre via www.ccdc.cam.ac.uk/data_request/cif.

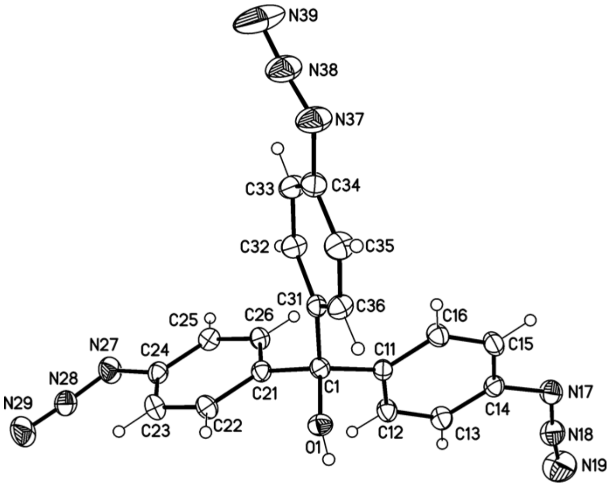

Figure S43. Molecular structure of **1** (displacement parameters are drawn at a 30 % probability level).

9. References

1. Bräse, S.; Gil, C.; Knepper, K.; Zimmermann, V., Organic Azides: An Exploding Diversity of a Unique Class of Compounds. *Angewandte Chemie International Edition* **2005,** *44* (33), 5188-5240.

2. Tat, J.; Heskett, K.; Satomi, S.; Pilz, R. B.; Golomb, B. A.; Boss, G. R., Sodium azide poisoning: a narrative review. *Clinical Toxicology* **2021,** *59* (8), 683-697.

3. Kutonova, K. V.; Trusova, M. E.; Postnikov, P. S.; Filimonov, V. D.; Parello, J., A Simple and Effective Synthesis of Aryl Azides via Arenediazonium Tosylates. *Synthesis* **2013,** *45* (19), 2706-2710.
